# Diffusion is capable of translating anisotropic apoptosis initiation into a homogeneous execution of cell death

**DOI:** 10.1186/1752-0509-4-9

**Published:** 2010-02-04

**Authors:** Heinrich J Huber, Maike A Laussmann, Jochen HM Prehn, Markus Rehm

**Affiliations:** 1 Sytems Biology Group, Department of Physiology & Medical Physics, Royal College of Surgeons in Ireland, Dublin 2, Ireland; 2 Siemens Ireland, Leeson Close, Dublin 2, Ireland

## Abstract

**Background:**

Apoptosis is an essential cell death process throughout the entire life span of all metazoans and its deregulation in humans has been implicated in many proliferative and degenerative diseases. Mitochondrial outer membrane permeabilisation (MOMP) and activation of effector caspases are key processes during apoptosis signalling. MOMP can be subject to spatial coordination in human cancer cells, resulting in intracellular waves of cytochrome-c release. To investigate the consequences of these spatial anisotropies in mitochondrial permeabilisation on subsequent effector caspase activation, we devised a mathematical reaction-diffusion model building on a set of partial differential equations.

**Results:**

Reaction-diffusion modelling suggested that even if strong spatial anisotropies existed during mitochondrial cytochrome c release, these would be eliminated by free diffusion of the cytosolic proteins that instantiate the apoptosis execution network. Experimentally, rapid sampling of mitochondrial permeabilisation and effector caspase activity in individual HeLa cervical cancer cells confirmed predictions of the reaction-diffusion model and demonstrated that the signalling network of apoptosis execution could efficiently translate spatial anisotropies in mitochondrial permeabilisation into a homogeneous effector caspase response throughout the cytosol. Further systems modelling suggested that a more than 10,000-fold impaired diffusivity would be required to maintain spatial anisotropies as observed during mitochondrial permeabilisation until the time effector caspases become activated.

**Conclusions:**

Multi-protein diffusion efficiently contributes to eliminating spatial asynchronies which are present during the initiation of apoptosis execution and thereby ensures homogeneous apoptosis execution throughout the entire cell body. For previously reported biological scenarios in which effector caspase activity was shown to be targeted selectively to specific subcellular regions additional mechanisms must exist that limit or spatially coordinate caspase activation and/or protect diffusing soluble caspase substrates from unwanted proteolysis.

## Background

Many signals initiating programmed cell death originate from specific subcellular sites or organelles, and thus require to be forwarded across intracellular space to trigger cellular suicide. Activated death receptors localize in distinct lipid raft micro domains in the plasma membrane for efficient formation of death inducing signalling complexes [1,2]. These sites represent spatially confined regions from which death signals may emanate, either in the form of activated initiator caspases-8/-10 which can directly activate effector caspase-3, or in the form of Bid, a pro-apoptotic cytosolic BH3-only protein of the Bcl-2 super family which is cleaved and activated by caspases-8/10 [3-5]. Similarly, BH-3 only proteins such as Bmf and Bim were shown to be associated to distinct cytoskeletal structures and are released during intrinsic apoptosis induced by various stimuli [6,7]. The cell death signals of these apoptosis inducers can be considered to spread through the cytosol by diffusion, analogous to diffusible signalling molecules in second messenger systems, and are integrated at the mitochondria, culminating in the permeabilisation of their outer membrane by pores comprised of activated Bax/Bak molecules [8]. Indeed, we and others recently showed in HeLa cervical cancer cells that apoptotic mitochondrial permeabilisation during extrinsic and intrinsic apoptosis can be subject to remarkable spatial coordination, resulting in waves of mitochondrial cytochrome-c (cyt-c) release progressing through the cell body [9,10]. Evidence has been provided also from other experimental systems that the process of MOMP can occur heterogeneously throughout the cell body [10,11]. Besides spatially inhomogeneous or polarized formation of caspase-8/-10 activation platforms during death receptor-induced apoptosis [1,12], kinase activities which may modulate the accessibility of caspase-8/-10 substrate Bid appear to play a role in regulating the spatial progression of mitochondrial permeabilisation [9].

Mitochondrial waves were also observed during intrinsic apoptosis induced by various other stimuli such as ceramide, staurosporine or direct pharmacological Bak activation [9-11]. Depending on the cell type investigated, spatial signal spread in these scenarios may at least in part depend on Ca^2+ ^signalling, kinase activities and/or also on additional, presently unknown signalling processes which also might determine the initiation site of MOMP. The spatial progression of mitochondrial permeabilisation therefore appears to be a common and frequent feature in multiple signalling paradigms during apoptosis initiation.

As mitochondrial Bax/Bak release pores were described to be rather non-selective [13], spatial MOMP waves can also be expected for other pro-apoptotic proteins besides cyt-c which are released from mitochondria, such as the XIAP antagonists Smac/DIABLO and HtrA2/Omi [14-16]. Once released, these proteins are able to initiate the activation of effector caspases, the main executioners of apoptosis.

Presently, it is not known whether the occurrence of spatial anisotropies in mitochondrial permeabilisation correlates with a spatially coordinated or targeted activation of effector caspases [17-19]. Previous single-cell imaging and mathematical modelling studies of apoptosis execution provided valuable insight into the temporal signalling kinetics and systems properties of the apoptosis execution network but ignored diffusion processes [20,21]. Building on this, we here therefore developed a reaction-diffusion model of the apoptosis execution network to investigate how protein diffusion impacts not only on the temporal but also on the spatial coordination of apoptotic cell death. This also served as an *in silico *estimation as to whether a functional link could exist between previously reported biological findings on spatially inhomogeneous apoptosis initiation and targeted or locally restricted of apoptotic executioner caspase activities [9-11,17-19].

## Results

### Generation of a reaction-diffusion model of apoptosis execution for HeLa cervical cancer cells

To investigate how spatial anisotropies during mitochondrial permeabilisation and cyt-c release reflect in the subsequent response of cytosolic effector caspase activation we performed an *in silico *analysis of the signalling dynamics using a spatial systems model. This model describes the reaction network of cyt-c initiated apoptosis execution according to a previously described network topology [21]. In the model, cyt-c and Smac release were implemented as processes independent of postmitochondrial feed-back by executioner caspases [22-25]. Released cyt-c then triggers apoptosome formation, whose absolute concentration was limited by the total amount of available procaspase-9 or Apaf-1 [21]. Cyt-c concentrations in this scenario were assumed not to limit apoptosome formation [26]. The kinetics of cyt-c induced apoptosome formation and Smac release were modelled with kinetics determined experimentally previously [21,25,27] (see Additional File 1).

From these inputs the model calculated the resulting effector caspase activation profile by assuming the following biochemical processes: Procaspase-9 bound to the apoptosome can auto-catalytically process itself to its p35/p12 form and can activate procaspases-3 and -7 by proteolysis [28,29]. Active caspase-3 can process caspase-9 to its p35/p10 form in positive feedback [30,31]. X-linked inhibitor of apoptosis protein (XIAP) was implemented as an inhibitor of caspase-9 (p35/p12) and caspases-3 and -7 [30,32]. In reverse, XIAP can be cleaved by caspase-3 into BIR1-2 and BIR3-RING cleavage fragments [33]. XIAP binding partners were assumed to be ubiquitinated and targeted for enhanced proteasomal degradation [34,35]. Effector caspase activity furthermore was implemented to impair protein synthesis and protein degradation [36,37]. Substrate cleavage by effector caspases represented the output of the model, allowing comparison of model outputs with experimental traces obtained from the cleavage of recombinant caspase substrates [21].

A non-spatial reaction network on the basis of mass action kinetics for the above delineated processes was already described previously as a set of ordinary differential equations (ODE) [21]. While this ODE model neglected diffusion processes, it was sufficient to reliably represent whole cell kinetics of apoptosis execution as experimentally recorded at minutes resolution in HeLa cervical and MCF-7 breast cancer cells [21]. As fast diffusion-adsorption processes likely contribute to the subcellular spatial spread of mitochondrial permeabilisation [9], we developed a spatial extension of the ODE model towards a reaction-diffusion network comprised of partial differential equations (PDEs).

**Figure 1:**
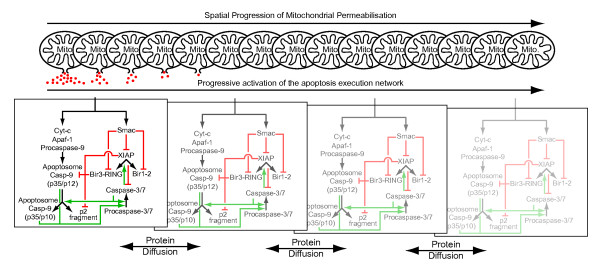
**Concept of implementing the reaction-diffusion model of apoptosis execution**. The model was implemented by assigning a spatially progressing permeabilisation of the outer mitochondrial membrane. Through membrane pores, Smac and cyt-c are released into the cytosol (red dots) where they initiate the apoptosis execution network. Cyt-c induces apoptosome formation, while Smac inhibits caspase inhibitor XIAP. As visualised here by multiple panes of the signalling map, the spatially progressing activation of the apoptosis execution network was modelled in discrete steps (n = 300) across distance (30 μm). The reaction network was calculated by partial differential equations and allowed for diffusive exchange of the reactants along spatial concentration gradients.

A graphical representation of the model is shown as Fig. 1. All state variables and associated reactions are provided in Additional File 1, Tables S1 and S2. To implement diffusion in the model, we assigned diffusion coefficients *D *to the individual proteins in the reaction network based on their molecular masses. The diffusion coefficients were calculated by the Stokes-Einstein relation in reference to the diffusion coefficient of a 27 kDa GFP protein which was previously determined as *D*_*GFP *_= 24 μm^2^s^-1 ^in cellular cytosolic environments [38]:(1)

The mass for all proteins and protein complexes as well as their calculated diffusion coefficients are listed in Additional File 1, Table S3.

Concentration changes ∂*c*_*n *_of the individual reacting proteins and protein complexes *n *by diffusion in time and space were implemented obeying Fick's second law for non-steady state diffusion in one dimension [39]. Modelling was performed in one spatial dimension to balance the abstraction of the biological scenario versus a mathematically manageable systems model (see also Methods). To qualitatively estimate the error incurring from the dimension reduction, we compared diffusive signal progression in one and three spatial dimensions for a simplified diffusion scenario (Additional File 2). The spatiotemporal discrepancies in diffusive spread between the two scenarios were very small and suggested that one-dimensional modelling could be employed to investigate the reaction-diffusion processes during apoptosis execution.

The network was initiated by two first order exponential saturation functions (see input functions in Additional File 1, Table S2), representing pseudo-reactions for cyt-c dependent apoptosome formation and Smac release into the cytosol. Highest possible spatial anisotropies of these two processes due to mitochondrial permeabilisation waves were implemented by modelling that apoptosis is initiated first at one end of a cell and then travels to the other end by assigning location-dependent onset times.(2)

with t_MOMP_(x_0_) = 0 at the point of origin x_0 _= 0 and *v*_*wave *_being the cellular end-to-end velocity of the spatial spread of mitochondrial permeabilisation [9].

Once initiated, each reaction partner *c*_*n *_distributes in the cytosol according the reaction-diffusion equation(3)

which represents the extension of Fick's second law for non-steady state diffusion by (*x*, *t*). This summand describes the biochemical reaction velocities of proteins and protein complexes *n *according to mass action kinetics (Additional File 1, Table S4). The modelled distance *L *was limited to 30 μm by assigning flux derivatives as zero at the near and far end boundaries as in Eq.4 and resembles the average length of HeLa cervical cancer cells.(4)

The calculations were discretized into 300 steps along the modelled distance. Analogous to the ODE model, the PDE model provided cleavage kinetics of a caspase-3 substrate as an output for comparison to experimentally obtained spatiotemporal data of caspase-3 substrate cleavage in single living cells.

### Mathematical modelling suggests that spatial anisotropies during apoptotic mitochondrial permeabilisation are lost upon activation of effector caspases

**Figure 2:**
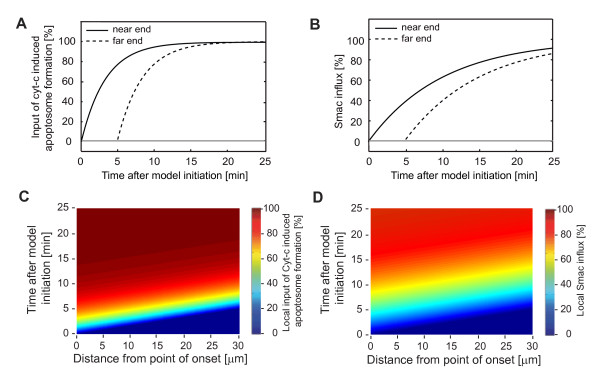
**Spatiotemporal initiation of the reaction-diffusion model of apoptosis execution**. **(A, B) **The distinct kinetics of the input functions for cyt-c induced apoptosome formation and Smac influx into the reaction network are shown for both the near and far ends of the modelled cell. **(C, D) **The local inputs of cyt-c induced apoptosome formation and Smac influx are shown for the full modelled distance. The pseudo-colour scales allow to read off how the local inputs proceed versus time. Identical colours display in slopes across distance and indicate that the initiation of both input functions progresses simultaneously. Smac influx follows a slower local input kinetic than cyt-c induced apoptosome formation, as visible by distinct colour gradients versus time for the two processes.

We initially set out to mathematically determine the influence of protein diffusion on the spatiotemporal activation profile of effector caspases. To resemble experimentally determined slow waves of apoptotic mitochondrial permeabilisation [9] (see Additional File 3 for a representative movie), the input functions for cyt-c induced apoptosome formation and Smac release were triggered with propagation velocities of 0.1 μm/s. For a modelled cell of 30 μm length the local inputs of cyt-c induced apoptosome formation and cytosolic Smac hence were delayed by 5 min between the near and far ends (Fig. 2A-B). According to previous experimental analyses [21,25], Smac release locally proceeded with slower kinetics than cyt-c induced apoptosome formation (Fig. 2A-D).

**Figure 3:**
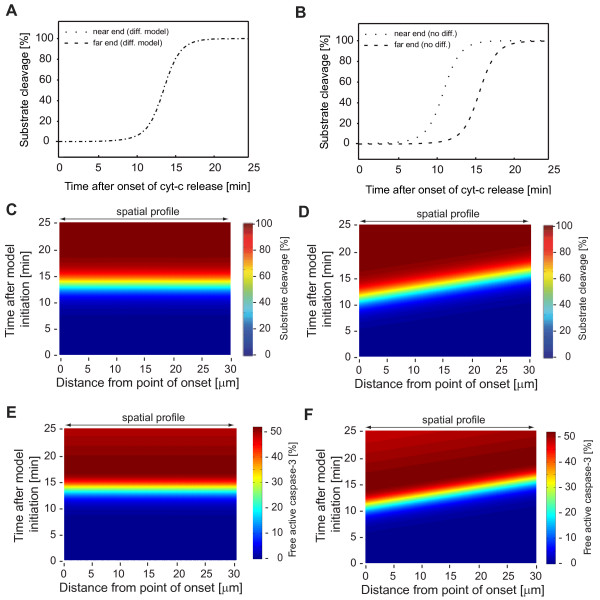
**Conversion of spatially anisotropic model inputs into a homogeneous activation of effector caspases**. **(A, B) **Kinetics of substrate cleavage by effector caspases at the near and far ends of the modelled cell in presence (A) or absence (B) of diffusion. In presence of diffusion, substrate cleavage kinetics superimpose at the near and far ends. **(C, D) **Full spatiotemporal profiles of substrate cleavage by effector caspases in presence (C) or absence (D) of diffusion. The pseudo-colour scale indicates percentage of substrate cleavage in space and time. Homogeneous substrate cleavage profiles across the full distance were observed in presence of diffusion, while delays displayed in absence of diffusion. **(E, F) **Full spatiotemporal profiles of free effector caspase-3 in presence (C) or absence (D) of diffusion. The pseudo-colour scale indicates the amount of free active caspase-3 as percentage of the total pro-caspase-3 present before initiating the reaction network.

When analyzing the resulting spatiotemporal profiles of substrate cleavage by effector caspases, the spatial anisotropies of the model input were translated into a homogeneous response across the modelled distance (Fig. 3A, C). In contrast, upon running the simulation in absence of diffusion, the spatiotemporal delays of the MOMP wave as expected were maintained (Fig. 3B, D). Corresponding results were obtained for the calculated amounts of free active effector caspase-3 in presence and absence of diffusion (Fig. 3E, F). Of note, protein diffusion caused a delay in effector caspase activity at the near end, and an earlier occurrence of caspase activity at the far end when compared to the model in absence of diffusion (Fig. 3E, F). Even though spatial waves of apoptotic permeabilisation of the outer mitochondrial membrane can be observed experimentally in HeLa cervical cancer cells, these simulations suggested that cytosolic diffusion may eliminate these spatiotemporal anisotropies during apoptosis execution.

### The elimination of spatial anisotropies during apoptosis execution is highly robust to impaired protein diffusivity

Protein diffusivity depends not only on particle size (molecular weight), but also on temperature and the viscosity of the intracellular microenvironment. These parameters cannot be meaningfully altered in experimental settings without massively compromising cellular physiology and vitality. Theoretical systems modelling therefore is particularly appropriate to evaluate the power of molecular diffusion in eliminating spatial anisotropies during apoptotic signalling.

**Figure 4:**
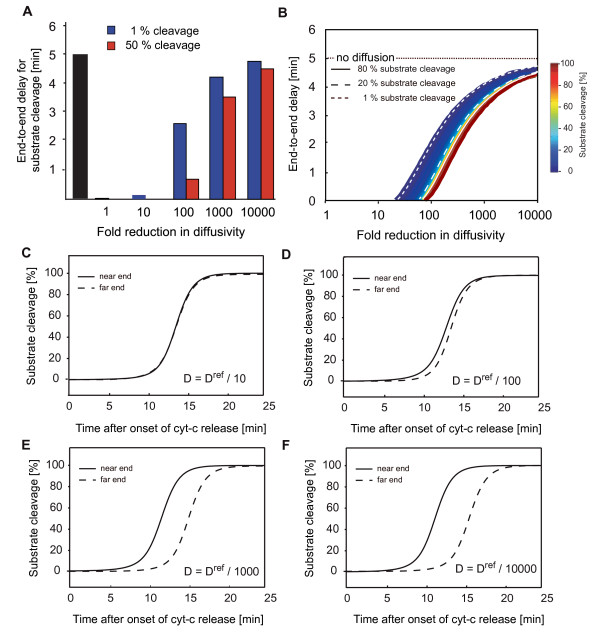
**A strong impairment of protein diffusivity is required to maintain spatial anisotropies during apoptosis execution**. **(A) **The end-to-end delays in achieving half maximal cleavage of effector caspase substrates has been calculated for the modelled HeLa cell scenario at impaired diffusion across 5 orders of magnitude. **(B) **End-to-end delays for the full spectrum of substrate cleavage across a continuous range of impaired diffusivities. **(C-F) **Temporal profiles of substrate cleavage at the near and far ends of the modelled cell for 10, 100, 1000, and 10000-fold impaired diffusivity.

We employed the *in silico *HeLa cell model to investigate to which extent diffusion needs to be impaired to allow for non-homogeneous signalling to persist from mitochondrial permeabilisation until effector caspase activation. We therefore recalculated the spatiotemporal signalling kinetics at reduced protein diffusivities and compared end-to-end delays in onset of substrate cleavage (1% cleavage) and half maximal substrate cleavage. Spatial asynchronies in substrate cleavage could be observed upon 100-fold reduction of diffusion, while 10,000-fold reduced diffusion coefficients were required to obtain end-to-end delays closely resembling conditions in the absence of diffusion (Fig. 4A). A subsequent, more detailed analysis confirmed that the early onset phase would be most prone to spatial anisotropies, with the onset of substrate cleavage by effector caspases becoming asynchronous upon 25-fold impaired diffusion (Fig. 4B). Of note, no major difference in end-to-end delays could be observed for higher amounts of cleaved substrate (Fig. 4B). Reducing diffusion by up to 4 orders of magnitude and plotting the full profiles of substrate cleavage at the near and far ends correspondingly showed the noticeable temporal separation of substrate cleavage profiles upon approx. 100-fold reduced diffusivity (Fig. 4C-F). These findings therefore demonstrate the strong influence of diffusion in eliminating spatial anisotropies within a closed cellular system.

To further explore the signalling system for processes that potentially contribute to or counteract a spatially synchronous execution of apoptosis, we investigated the role of caspase-dependent feed-backs, altered reaction kinetics and the consequences of immobilising macromolecular aggregates.

### The role of caspase-dependent feed-backs in spatiotemporal apoptotic signalling

**Figure 5:**
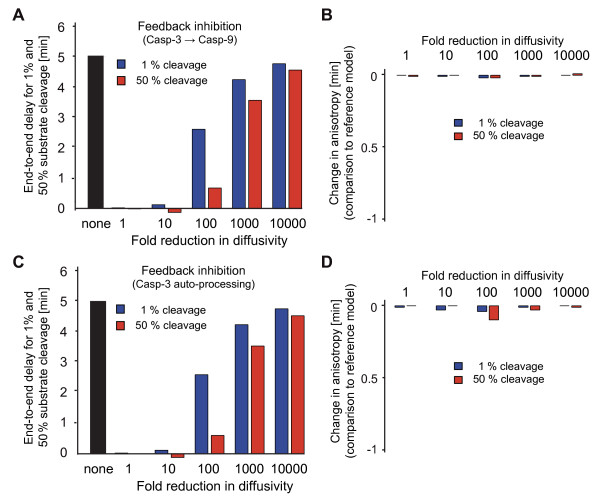
**Consequences of impaired caspase feed-back on spatial anisotropies during apoptosis execution**. **(A) **The end-to-end delays in onset of substrate cleavage (1%) as well as the delays for half maximal substrate cleavage by effector caspases were calculated in absence of positive feedback from caspase-3 onto caspase-9 p35/p12. In parallel, diffusivity was modified across 5 orders of magnitude. **(B) **End-to-end delays from the non-perturbed reference model were subtracted from the results displayed in (A). Negative values represent reduced spatial anisotropies due to inhibition of feed-back of caspase-3 onto caspase-9 p35/p12. **(C) **The end-to-end delays in onset of substrate cleavage (1%) as well as the delays for half maximal substrate cleavage by effector caspases were calculated in absence of caspase-3 auto-processing. In parallel, diffusivity was modified across 5 orders of magnitude. **(D) **End-to-end delays from the non-perturbed reference model were subtracted from the results displayed in (C). Negative values represent a reduction in spatial anisotropies due to inhibition of caspases-3 auto-processing.

Systems analysis of the MAPK pathways recently suggested feed-back circuits to contribute to the rapid spatial progression of phosphoprotein signals [40]. In analogy, feed-back amplification loops during apoptosis execution, such as detected between caspase-3 and caspase-9, likewise could contribute to the swift spatial spread of death signals. To obtain further insight into the role of caspase feed-back for a spatially homogeneous response of apoptosis execution in the HeLa cell systems model, we investigated scenarios in which either the caspase-3 feedback onto caspase-9 (p35/p12) or the capase-3 auto-processing were blocked. Eliminating these feedbacks resulted in reduced amounts of processed caspases, an overall delay in substrate cleavage (not shown), and comparable end-to-end anisotropies in substrate cleavage between both scenarios (Fig. 5A, C). If caspase feed-backs had a role in promoting spatial anisotropies, we expected their inhibition to result in a reduction of end-to-end delays when compared to the reference model (see Fig. 4A). Reductions could indeed be observed (Fig. 5B, D), however these were rather modest if not negligible.

### Increased biochemical reactivity and immobilisation of apoptosome species promote spatial anisotropies

**Figure 6:**
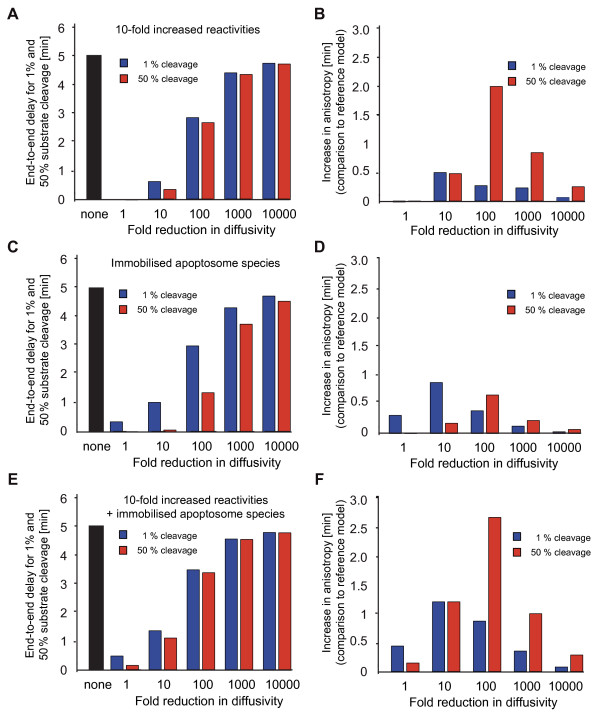
**Consequences of altered reaction rates and apoptosome immobilisation on spatial anisotropies during apoptosis execution**. **(A, B) **Acceleration of biochemical reactions promotes maintenance of spatial anisotropies during apoptosis execution. **(A) **The end-to-end delays in onset of substrate cleavage (1%) as well as the delays for half maximal substrate cleavage by effector caspases were calculated for a 10-fold increase in biochemical reaction rates. In parallel, diffusivity was modified across 5 orders of magnitude. **(B) **End-to-end delays from the non-perturbed reference model were subtracted from the results displayed in (A). Positive values represent an increase in spatial anisotropy. **(C, D) **Immobilized apoptosomes promote spatial anisotropies during apoptosis execution. **(C) **Assuming immobile apoptosome complexes, the end-to-end delays in onset of substrate cleavage (1%) as well as the delays for half maximal substrate cleavage by effector caspases were calculated. In parallel, diffusivity was modified across 5 orders of magnitude. **(D) **End-to-end delays from the non-perturbed reference model were subtracted from the results displayed in (C). Positive values represent an increase in spatial anisotropy. **(E, F) **Influence of a combination of accelerated biochemical reactions and macromolecule immobilisation on spatial anisotropies during apoptosis execution. **(E) **The end-to-end delays in onset of substrate cleavage (1%) as well as the delays for half maximal substrate cleavage by effector caspases were calculated. In parallel, diffusivity was modified across 5 orders of magnitude. **(F) **End-to-end delays from the non-perturbed reference model were subtracted from the results displayed in (E). Positive values represent an increase in spatial anisotropy.

The rate constants for the reactions included in our systems model largely were determined by quantitative biochemical experimentation using purified reactants at low total protein concentrations in aqueous reaction buffers (see Additional File 1 for references). Reaction rates in macromolecule-rich environments such as the cytosol can substantially differ from those observed *in vitro *due to the volume exclusion arising from macromolecular crowding [41]. Volume exclusion therefore can potentially accelerate the kinetics of protein-protein interactions and enzymatic catalyses, given that the reactants are present in sufficient abundance [41]. We therefore repeated the preceding calculations of end-to-end delays in substrate cleavage (Fig. 4A) in a variant of the HeLa cell model that employed 10-fold accelerated reaction kinetics. While substrate cleavage at normal diffusivity still proceeded synchronously throughout the cell, end-to-end delays now became apparent upon ≥ 10-fold reduced diffusivities (Fig. 6A). Comparison of the end-to-end delays to the reference model highlighted that anisotropies in the onset of substrate cleavage (1% cleavage) were only mildly affected, whereas higher spatial heterogeneities displayed for half-maximal substrate cleavage (Fig. 6B).

The diffusive mobility of very large proteins and protein complexes (≥500 kDa) was shown to be significantly impaired in dense cytosolic environments containing microcompartments [42] and could thereby promote spatial heterogeneities. We therefore also simulated a scenario in which the apoptosome (approx. 700 kDa) and all apoptosome-bound protein species were considered immobile (Fig. 6C). In this case, anisotropy discrepancies to the reference model were most pronounced for the onset of substrate cleavage (Fig. 6D). A combination of 10-fold increased biochemical reactivity and apoptosome immobilisation further enhanced the calculated spatial heterogeneities (Fig. 6E) and resulted in considerable anisotropies both in the onset of substrate cleavage as well as in half-maximal substrate cleavage (Fig. 6F). As expected, in all scenarios discrepancies to the reference model were rather small at strongly impaired diffusivity (10,000-fold) (Fig. 6B, D, F), as for this condition high spatial anisotropies already existed in the reference scenario (Fig. 4A).

Taken together these data indicate that acceleration of biochemical reactions arising from volume exclusion as well as immobilisation of large macromolecular aggregates such as the apoptosome could promote spatial anisotropies during apoptosis execution.

### Experimental validation of model prediction: Spatial anisotropies during mitochondrial permeabilisation are eliminated upon apoptosis execution in HeLa cervical cancer cells

**Figure 7:**
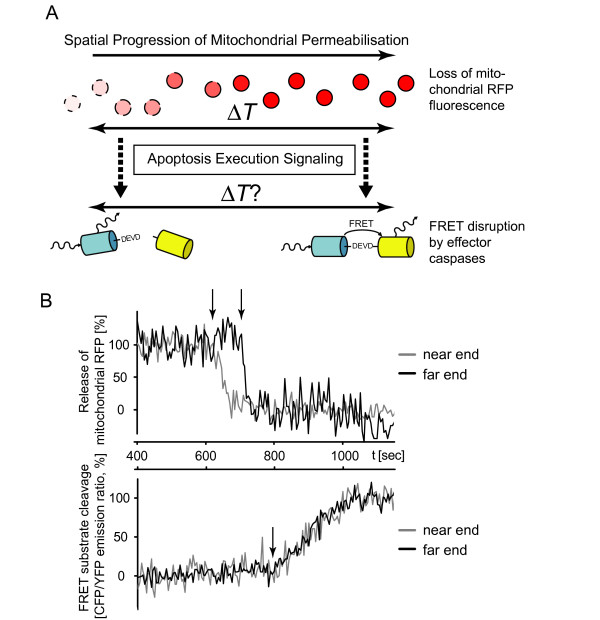
**Experimental validation of the model prediction: Elimination of spatial anisotropies during apoptosis execution**. **(A) **Design of the experimental approach. The release of red fluorescent protein from mitochondria was determined in individual cells. The region of the cell that showed the release first was defined as the near end, whereas the far end represented the side of the cell that the signal travelled to. In parallel, the cleavage of a recombinant effector caspase FRET substrate (CFP-DEVD-YFP) was investigated. Upon cleavage of the FRET probe the blue emission increases, while in the intact probe resonance energy transfer allows for YFP emission upon CFP excitation. The delays between onset of RFP or FRET probe cleavage between near and far ends were determined. **(B) **Representative traces from a HeLa cervical cancer cell analysed by rapid sampling of apoptotic signalling. The cell was treated with 100 ng/ml TRAIL/1 μg/ml CHX. Mitochondrial permeabilisation was measured by RFP release while effector caspase activation was measured by CFP-DEVD-YFP FRET disruption in regions at the near and far ends of the cell. Black arrows indicate onset of the respective events. The delay in mitochondrial permeabilisation is lost upon FRET substrate cleavage. 4 cells with corresponding results were obtained from n = 4 experiments.

We next validated experimentally in HeLa cervical cancer cells whether effector caspase activity sets on synchronously throughout the cytosol. To this end, we combined mitochondrial protein redistribution measurements with a FRET-based analysis of effector caspase activity in individual cells. The FRET probe consists of cyan fluorescent protein (CFP) and yellow fluorescent protein (YFP) interconnected by a short linker containing the preferred caspase-3 recognition site DEVD [43,44]. HeLa cells expressing the DEVD FRET probe were co-transfected with a plasmid coding for expression of a monomeric red fluorescent protein targeted to the mitochondrial intermembrane space (IMS-RP). This probe was previously shown to be released from the mitochondria into the cytosol similarly to cytochrome-c and Smac during MOMP [45] and served to identify mitochondrial permeabilisation waves (Fig. 7A). Imaging was performed by employing a previously described technique that allowed rapid sampling in absence of noticeable photobleaching or phototoxicity [9]. We achieved a temporal resolution of as little as 4 sec when sampling CFP, FRET, YFP, and RFP channels for individual cells, representing a 30-fold higher temporal resolution than previous time-lapse imaging studies. The temporal resolution could not be further increased due to the time required for physically moving the optical components in the instrument to acquire the different channels.

Following apoptosis induction with 100 ng/ml TRAIL/1 μg/ml CHX, we observed temporal delays in the onset of IMS-RP release from mitochondria into the cytosol between the near and far ends of individual cells, thus confirming that mitochondrial permeabilisation could proceed anisotropically (Fig. 7B, representative for n = 4 experiments). In contrast, effector caspase activation always occurred simultaneously in both regions (Fig. 7B).

Lipotransfection to achieve sufficiently high expression of the IMS-RP was accompanied by some spontaneous cell death of HeLa cells. To exclude cellular stress as a factor influencing the experimental results, we aimed to further validate our finding in a larger number of HeLa cells without expression of this probe. We previously identified that in response to 1 μM STS or TRAIL/CHX (100 ng/ml/1 μg/ml) spatial anisotropies of mitochondrial permeabilisation can be detected in up to 70% of cells [9]. Following STS or TRAIL/CHX treatments, individual cells were analyzed for effector caspase activation in regions at opposite ends of the cell body. In all cells analyzed (n = 14 in response to STS and n = 20 cells in response to TRAIL/CHX) FRET probe cleavage started simultaneously at opposite ends of individual cells (see Additional File 4). These experimental data therefore confirm the prediction if the reference model that spatial anisotropies during the initiation of mitochondrial permeabilisation are efficiently translated into a homogeneous response of effector caspase activation throughout the entire cytosol.

## Discussion

Our mathematical estimation of the spatial dynamics during apoptosis execution suggests that diffusion of the multiple proteins and protein complexes involved in the execution network would equalize any spatial anisotropies that were initially present during the time period required from cyt-c release to effector caspase-3 activation. Fast sampling of effector caspase activation confirmed experimentally that even though MOMP can be spatiotemporally organized, the signalling network during apoptosis execution translates this into a homogeneous response of effector caspase activation.

The mathematical reference model we devised is simplistic in that it omits the explicit consequences of macromolecular crowding and diffusion barriers: With respect to diffusion the cytosol cannot be considered to be a homogeneous aqueous environment as it is densely crowded by organelles and macromolecules. The macro-molecularly excluded volume was estimated as 20-30%, resulting in up to 3-fold reduced diffusion coefficients for both small and large molecules in eukaryotic cells [46]. As our calculations of protein diffusion built on a reference diffusion coefficient for green fluorescent protein measured in an intracellular environment [38], these factors can be considered to be taken into account at least partially, while impaired diffusivity of very large protein complexes that may violate the Stokes-Einstein relation were investigated separately (Fig. 6).

We found that lack of caspase feedbacks had negligible influence on spatiotemporal asynchronies during apoptosis execution, which might seem counterintuitive given that feed backs in other scenarios were shown to play a significant role in the spatial spread of protein signals [40]. As absence of caspase feedbacks results in an overall slower signalling during apoptosis execution, the prolonged lag time between MOMP and substrate cleavage allows for diffusion to efficiently eliminate spatial anisotropies.

Of note, our modelling was performed assuming one-dimensional reaction-diffusion processes as anisotropic three-dimensional scenarios currently cannot be handled mathematically by the available modelling tools. Even though for a simplified scenario of one diffusing protein largely identical spatiotemporal spreads were calculated (Additional File 2), we would expect that for biologically more authentic cases of three-dimensional, non-symmetric adherent cells further distortions between both scenarios could become apparent. The mathematical model also assumes strict location-dependent onset times of mitochondrial permeabilisation, whereas it can be conceived that in a living cell system the propagation process might naturally be subject to variability. Furthermore, additional signalling events may exist that affect the spatial spread of signalling in different cell types or cells lines and which are not covered in our model. Nevertheless, the one-dimensional modelling approach still was sufficiently accurate to predict the subsequent experimental finding that spatial anisotropies would be eliminated at standard conditions in HeLa cells.

As shown in this study by modelling impaired diffusivity, more than 100-fold lowered diffusion coefficients were required to maintain spatial anisotropies during apoptosis execution in the reference scenario. In many signal transduction systems the activation and deactivation of the respective signalling molecules are spatially separated to establish or maintain spatial anisotropies in presence of diffusion, such as for example in the epidermal growth factor receptor/phosphotyrosine phosphatase 1B and the mitogen-activated protein kinase (MAPK) signalling systems [47,48]. When assuming that both upstream and downstream proteins during apoptosis execution are not locally retained or produced/degraded, it seems reasonable that spatial anisotropies can only be maintained upon strong impairment of diffusivity. In addition, cytoplasmic agitation by molecular motors was recently suggested to further enhance cytosolic molecular motions and thus to promote the efficiency of spatial homogenisation of otherwise solely diffusive processes [49].

While diffusion can ensure the efficient execution of cell death during canonical apoptotic signalling, several studies suggested that in certain scenarios effector caspase activity can be targeted selectively to specific subcellular regions and to limited sets of substrates. For example, effector caspase activity targeted exclusively towards the nucleus seems to be required for the differentiation of lens cells, erythrocytes, and megakaryocytes [17-19]. Our data would suggest that in these particular situations additional mechanisms must exist that (i) limit and confine active caspases to target regions and/or (ii) protect diffusing soluble caspase substrates from unwanted proteolysis. Localised accumulation or compartmentalisation of active caspases by as of yet unknown anchoring mechanisms indeed has been reported before [17]. However, as literally hundreds of known protein substrates, many of them soluble, can be cleaved by caspases [50,51] the control of local, substrate-selective caspase activity must be subject to further control mechanisms. Additional levels of control could rely on regulating caspase activities and substrate availabilities by (reversible) posttranslational modifications. The susceptibilities of several caspase substrates were previously shown to be modulated by phosphorylation, as were the activities of caspases-9 and 3 themselves [52,53], suggesting that kinase/phosphatase signalling could be crucial for both direct caspase regulation as well as for restricting or specifying substrate availabilities by modulating the cellular phosphoproteome. As recently shown for the *Drosophila *effector caspase drICE, a homologue of human effector caspases-3 and -7, ubiquitin conjugation could likewise modulate activities by impairing substrate access to the catalytic site of the enzyme [54]. In the light of the rapidness of multi-protein diffusion during apoptosis execution, our study therefore also highlights the physiological significance of these control mechanisms, which so far have been largely ignored in spatially restricted, sublethal caspase activation scenarios.

## Conclusions

Our study highlights that diffusion of the multiple proteins constituting the apoptosis execution network is sufficient to robustly eliminate the spatial asynchronies that can be observed during the initiation of apoptosis. It is therefore highly unlikely that anisotropic initiation of apoptosis is linked mechanistically to scenarios in which effector caspase activities were reported to be subcellularly coordinated or confined. A homogeneous activation of effector caspases throughout the entire cell body due to diffusion might indeed be an important contributor to the efficiency of apoptotic cell clearance.

## Methods

### Model Implementation

The reaction-diffusion model was implemented in MATLAB (The Mathworks, UK) for numerical analysis. Partial differential equations were solved using the PDEPE subroutine which uses an adaptive step Runge-Kutta ODE solver [Gear74]. The model code is available as a Additional File 5 to this manuscript.

Modelling was performed in one spatial dimension, as solvers for partial differential equations for a 4D analysis of three spatial and one temporal dimension and anisotropic inputs are not available for MATLAB. An error estimation for diffusive signal progression in one and three spatial dimensions was performed for a simplified diffusion scenario (Additional File 2).

### Materials

Embryo-tested mineral oil and cycloheximide (CHX) were from Sigma (Tallaght, Ireland). TRAIL was from Leinco Technologies (St. Louis, MO, USA). TMRM was from MobiTec (Göttingen, Germany).

### Cell culture

HeLa cells were cultured in RPMI 1640 medium supplemented with penicillin (100 U/ml), streptomycin (100 μg/ml) and 10% fetal calf serum (Sigma, Tallaght, Ireland). Cells were transfected with plasmid DNA (pmyc-CFP-DEVD-YFP or p-IMS-RP) as described previously [43,45].

### Microscopy and image analysis

Imaging was performed using a Zeiss LSM 510 META inverted confocal microscope (Carl Zeiss, Germany) using a previously developed rapid sampling approach building on the mitotic history of sibling cells [9]. HeLa cells expressing the CFP-DEVD-YFP FRET probe alone or together with the IMS-RP probe were incubated over night to allow for sufficient cell division. Using a 63 × oil objective (N.A. 1.4), CFP and FRET signals were recorded upon excitation at 405 nm in a first scan, YFP and RFP signals were recorded upon excitation at 488 nm with a second scan. Fluorescence was monitored at zoom 0.7 using optimized filter and mirror sets for the fluorophores in the respective scanning steps. Upon drug addition, the full field of view was scanned at 2 min intervals. Upon apoptotic morphology (cellular shrinkage, blebbing) of an individual cell, the scan area was reduced to include the respective sibling cell. Cells were then scanned for 20-30 min in reverse scan mode at intervals of as little as 4 sec (line step 4). As cellular geometry dictated the scan area, the intervals slightly varied between experiments. Untreated cells served as controls. Photobleaching could not be observed in the individual channels in control experiments (Additional File 6).

Onset of IMS-RP release from the mitochondrial intermembrane space was detected as a decrease in fluorescence intensity in subcellular mitochondrial regions as described previously for the release of cyt-c-GFP [9]. CFP-DEVD-YFP substrate cleavage was detected by fluorescence resonance energy transfer (FRET) analysis [43]. CFP/YFP emission ratio traces were obtained by dividing the fluorescence intensity values of subcellular regions after background subtraction. Microscopy images were processed using MetaMorph 7.0 (Molecular Devices, UK).

## Authors' contributions

HH: model design and implementation, data interpretation, writing the manuscript; ML: acquisition of data, analysis and interpretation of data; JP: coordination of the study, writing the manuscript; MR: conception, design and coordination of the study, acquisition of data, data analysis and interpretation, writing the manuscript. All authors read and approved the final manuscript.

## Supplementary Material

Additional file 1 **Modelling parameters for the reaction-diffusion model**. These additional tables list the state variables of the model (Table S1), the individual reactions and reaction constants including literature references (Table S2), molecular masses of all reactants (Table S3), and the biochemical reaction rates (Table S4). Click here for file

Additional file 2**Comparison of diffusive signal spread between simplified one and three dimensional scenarios**. Comparison of signal spread between simplified one and a three dimensional spatial models. The one dimensional model represents a linear slab with the input pulse located at the left boundary **(A)**. The three dimensional model represents a sphere with the input signal starting synchronously on the entire surface of the sphere **(B)**. Signal progression along the slab (1 dimensional model) or towards the centre of the sphere (3 dimensional model) was investigated. To mathematically handle the 3-dimensional spherical model with the PDEPE subroutine in MATLAB, the diffusion process was transformed to a problem of one spatial and temporal component without loss of information. This yielded the following reaction diffusion equation for the radial component  of species *n*:where *r *is the radius and *D*_*n *_denotes the diffusion coefficient. Only small discrepancies were observed between the two scenarios. More complex scenarios such as spatially anisotropic triggers could not be subjected to this dimension reduction.Click here for file

Additional file 3 **Spatial progression of mitochondrial permeabilisation**. A movie of a representative HeLa cervical cancer cell expressing a red fluorescent reporter protein targeted to the mitochondrial intermembrane space (IMS-RP) is shown. Release of IMS-RP results in a drop in fluorescence intensity. Following treatment with 1 μg/ml TRAIL/CHX, IMS-RP release is first initiated on the left side and progresses through the cell body until the release is complete. The movie represents a duration of 5.25 min. Click here for file

Additional file 4 **Spatially homogeneous caspase activation in HeLa cervical cancer cells**. Substrate cleavage by effector caspases was experimentally measured by CFP-DEVD-YFP FRET disruption at fast sampling rates in HeLa cells. FRET disruption was measured in regions at distal ends of the cell. The traces shown were obtained from a cell treated with 100 ng/ml TRAIL/1 μg/ml CHX. Arrow indicates onset of substrate cleavage. Corresponding results were obtained from n = 19 additional cells treated with TRAIL/CHX and n = 14 cells treated with 1 μM STS. Click here for file

Additional file 5 **MatLab script of the reaction-diffusion model**. The file contains the MatLab code for the reaction-diffusion model and the required annotations to repeat all modelling presented in this study. The model could not be provided as SBML as spatiotemporal PDE models are not yet supported. Click here for file

Additional file 6 **Photobleaching control measurement for rapid FRET sampling**. Fluorescence signal intensities in CFP, FRET, and YFP channels were measured in unstimulated cells at rapid sampling conditions. This control excludes that photodamage by the acquisition process influenced the experimental measurements of effector caspase activation. Click here for file
