# Supplementary material for: Diffusion is capable of translating anisotropic apoptosis initiation into a homogeneous execution of cell death
Source: BMC Syst Biol. 2010 Feb 4;4:9. doi: 10.1186/1752-0509-4-9 (PMC2831829; doi:10.1186/1752-0509-4-9)
Supplement: Additional file 1 — Modelling parameters for the reaction-diffusion model. These additional tables list the state variables of the model (Table S1), the individual reactions and reaction constants including literature references (Table S2), molecular masses of all reactants (Table S3), and the biochemical reaction rates (Table S4). [file 1752-0509-4-9-S1.DOC]

## Supplementary Tables

## Table S1: State variables of the mathematical HeLa cell model that represent momomeric and multimeric entities. Comments provide additional information for the respective parameters where required. Quantities of procaspase-3, XIAP, and procaspase-9 were determined directly or indirectly before [1].

| **Symbolic Abbreviation** | **Explanation** | **Initial value [nM]** | **Comment** |
| --- | --- | --- | --- |
| C3 | Procaspase-3 | 120 |  |
| C9a | Apoptosome associated caspase-9 (p35/p12) | 0 |  |
| C3a | Free active Caspase-3 | 0 |  |
| C9H | Apoptosome associated caspase-9 processed (p35/p10) | 0 |  |
| XIAP | Free x-linked Inhibitor of Apoptosis Protein | 63 |  |
| XIAP~C3a | XIAP in complex with caspase-3 | 0 |  |
| XIAP~C9a | XIAP in complex with apoptosome associated caspase-9 (p35/p12) | 0 |  |
| XIAP~C9a~C3a | XIAP in complex with both caspase-3 and apoptosome associated caspase-9 (p35/p12) (not used in current calculations) | 0 |  |
| XIAP~{cl-C9a} | XIAP in complex with the caspase-9 (p35/p12) derived p2 fragment | 0 |  |
| XIAP~{cl-C9a}~C3a | XIAP in complex with both caspase-3 and the p2 fragment | 0 |  |
| BIR12 | XIAP fragment comprising baculoviral IAP repeats 1 and 2 | 0 |  |
| BIR3R | XIAP fragment comprising baculoviral IAP repeat 3 and RING domain | 0 |  |
| BIR12~C3a | XIAP Bir12 fragment in complex with caspase-3 | 0 |  |
| BIR3R~C9a | XIAP Bir3R fragment in complex with apoptosome associated caspase-9 (p35/12) | 0 |  |
| BIR3R~{cl-C9a} | BIR3R bound to p2 fragment | 0 |  |
| SMAC | Second mitochondria-derived activator of caspases, dimer | 0 |  |
| XIAP~2SMAC | Smac dimer in complex with XIAP | 0 |  |
| XIAP~{cl-C9a}~ 2SMAC | XIAP in complex with SMAC dimer and p2 fragment | 0 |  |
| BIR12~SMAC | Smac dimer in complex with the XIAP Bir12 fragment | 0 |  |
| BIR3R~SMAC | Smac dimer in complex with the XIAP Bir3R fragment | 0 |  |
| Substrate | DEVD effector caspase FRET substrate | 100 % |  |
| C9Inact | Procaspase-9 | 30 |  |
| Smacmito | Mitochondrial Smac | 126 | Assumed to equal 2*XIAP |

Table S2: Reaction network for apoptosis execution initiated by mitochondrial permeabilisation. Input functions were modelled as first order reactions to resemble exponential saturation of cyt-c induced apoptosome formation and Smac release into the cytosol according to experimentally determined kinetics. Protein turnover of procaspase-3 and XIAP was adjusted to yield cytosolic concentrations of 120 nM and 63 nM, respectively. All other reactions represent catalytic cleavage, binding/dissociation reactions, or protein degradation. An extensive description of this reaction network was published before [1]. Applying mass action kinetics yields the reaction rates *v^reaction^_n_ (x,t)* for each reaction as listed in Supplementary Table 4.

| **Substrates** |  | **Products** | ***K_on_/K_cat_*** | ***K_off_*** | **Citation** |
| --- | --- | --- | --- | --- | --- |
| **Input functions** | | | **µM^-1^min^-1^** | **------** |  |
| C9Inact | → | C9a (cyt-c induced apoptosome formation) | Log(2)/2.3 | 0 | [1] |
| SMACmito | → | Smac (Smac release) | Log(2)/7 | 0 | [2] |
| **Apotosome dependent caspase activation** | | | **µM^-1^ min^-1^** | **--------** |  |
| C9a + C3 | → | C9a + C3a | 6 | 0 | [3] |
| C9a + C3a | → | C9H + C3a | 12 | 0 | [4] |
| C9H + C3 | → | C9H + C3a | 48 | 0 | [5] |
| **Caspase 3 autoprocessing** | | | **µM^-1^ min^-1^** | **--------** |  |
| C3 + C3a | → | 2 C3a | 2.4 | 0 | [5] |
| **XIAP inhibition of Caspases** | | | **µM^-1^ min^-1^** | **min^-1^** |  |
| C3a + XIAP | ↔ | XIAP~C3a | 156 | 0.144 | [6] |
| C3a + XIAP~{cl-C9a} | ↔ | XIAP~{cl-C9a}~C3a | 156 | 0.144 | [6] |
| C3a + BIR12 | ↔ | BIR12~C3a | 156 | 0.144 | [6] |
| C9a + XIAP | ↔ | XIAP~C9a | 156 | 0.144 | [6] |
| **Caspase cleavage of XIAP fractions** | | | **µM^-1^ min^-1^** | **--------** |  |
| C3a + XIAP | → | C3a + BIR3R + BIR12 | 12 | 0 | [4] |
| C3a + XIAP~C9a | → | C3a + BIR3R~C9a + BIR12 | 12 | 0 | [4] |
| C3a + XIAP~C3a | → | C3a + BIR12~C3a + BIR3R | 12 | 0 | [4] |
| C3a + XIAP~{cl-C9a} | → | C3a + BIR12 + BIR3R~{cl-C9a} | 12 | 0 | [4] |
| C3a + XIAP~{clC9a}~C3a | → | C3a + BIR12~C3a + BIR3R~{cl-C9a} | 12 | 0 | [4] |
| C3a + XIAP~2SMAC | → | C3a + BIR12~SMAC + BIR3R~SMAC | 12 | 0 | [4] |
| C3a + XIAP~C9a | → | C3a + C9H + XIAP~{cl-C9a} | 12 | 0 | [4] |
| C3a + BIR3R~C9a | → | C3a + BIR3R~{cl-C9a} + C9H | 12 | 0 | [4] |
| **Smac inihibition of XIAP** | | | **µM^-2^ min^-1^** | **min^-1^** |  |
| XIAP + 2 SMAC | ↔ | XIAP~2SMAC | 420 | 0.133 | [7] |
| XIAP~{cl-C9a} +2 SMAC | ↔ | XIAP~{cl-C9a} ~ 2SMAC | 420 | 156 | [7] |

| **Smac inihibition of XIAP fragments** | | | **µM^-1^min^-1^** | **min^-1^** |  |
| --- | --- | --- | --- | --- | --- |
| BIR12 + SMAC | ↔ | BIR12~SMAC | 4.45 | 31.9 | [7] |
| BIR3R + SMAC | ↔ | BIR3R~SMAC | 0.33 | 14.2 | [7] |
| **Smac inihibition of XIAP with caspase competition** | | | **µM^-2^min^-1^** | **min^-1^** |  |
| XIAP~C9a + 2 SMAC | ↔ | XIAP~2SMAC + C9a | 420 | 156 | combined from above |
| XIAP~C3a + 2 SMAC | ↔ | XIAP~2SMAC + C3a | 420 | 156 | combined from above |
| **Smac inihibition of XIAP fragments with caspase competition** | | | **µM^-1^min^-1^** | **min^-1^** |  |
| BIR12~C3a + SMAC | ↔ | BIR12~SMAC + C3a | 0.33 | 156 | combined from above |
| BIR3R~C9a + SMAC | ↔ | BIR3R~SMAC + C9a | 420 | 156 | combined from above |
| **Degradation reactions** | | | **min^-1^** | **------** |  |
| C9H | → | degradation | 0.0058 | 0 | [8] |
| C9a | → | degradation | 0.0058 | 0 | [8] |
| C3a | → | degradation | 0.0058 | 0 | [8] |
| XIAP~C3a | → | degradation | 0.0347 | 0 | [9] |
| XIAP~C9a | → | degradation | 0.0347 | 0 | [9] |
| XIAP~{cl-C9a} | → | degradation | 0.0058 | 0 | [8] |
| XIAP~{cl-C9a}~C3a | → | degradation | 0.0058 | 0 | [8] |
| XIAP~{cl-C9a}~SMAC | → | degradation | 0.0347 | 0 | [9] |
| XIAP~2SMAC | → | degradation | 0.0347 | 0 | [9] |
| BIR12 | → | degradation | 0.0058 | 0 | [8] |
| BIR3R | → | degradation | 0.0347 | 0 | [9] |
| BIR12~SMAC | → | degradation | 0.0058 | 0 | [8] |
| BIR3R~SMAC | → | degradation | 0.0347 | 0 | [9] |
| BIR12~C3a | → | degradation | 0.0058 | 0 | [8] |
| BIR3R~C9a | → | degradation | 0.0058 | 0 | [8] |
| BIR3R~{cl-C9a} | → | degradation | 0.0347 | 0 | [9] |
| SMAC | → | degradation | 0.0058 | 0 | [8] |
| **Substrate cleavage** | | | **µM^-1^min^-1^** | **------** |  |
| Substrate + C3a | → | C3a | 12 | 0 | [4] |
|  | | |  |  |  |
|  |  |  |  |  |  |
|  |  |  |  |  |  |

Table S3: Molecular masses for state variables as used for the calculation of diffusion properties. Masses were determined as indicatedin the row "mass calculation": (1) calculated protein masses for monomers, (2) apparent mass of the apoptosome [10], (3) hetero-oligomer masses calculated from monomeric entities, (4) masses calculated from the apparent apoptosome mass and apoptosome-bound monomeric entities, (5) proteins present as cytosolic dimers and (6) fixed input signals.

| **Symbolic abbreviation** | **Symbol in Table 4** | **Apparent mass [kD]** | **Mass calculation** |
| --- | --- | --- | --- |
| C3 | c1 | 32 | 1 |
| C9a | c2 | 700 | 2 |
| C3a | c3 | 58 | 5 |
| C9H | c4 | 686 | 4 |
| XIAP | c5 | 57 | 1 |
| XIAP~C3a | c6 | 172 | 3 |
| XIAP~C9a | c7 | 1099 | 4 |
| XIAP~C9a~C3a | c8 | 760 | 4 |
| XIAP~{cl-C9a} | c9 | 59 | 3 |
| XIAP~{cl-C9a}~C3a | c10 | 176 | 3 |
| BIR12 | c11 | 28 | 1 |
| BIR3R | c12 | 29 | 1 |
| BIR12~C3a | c13 | 114 | 3 |
| BIR3R~C9a | c14 | 896 | 4 |
| BIR3R~{cl-C9a} | c15 | 31 | 3 |
| SMAC | c16 | 27 | 1 |
| XIAP~2SMAC | c17 | 111 | 3 |
| XIAP~{cl-C9a}~ 2SMAC | c18 | 113 | 3 |
| BIR12~SMAC | c19 | 82 | 3 |
| BIR3R~SMAC | c20 | 83 | 3 |
| C9Inact | c22 | Spatial Input (Eq.3) | 6 |
| Smacmito | c23 | Spatial Input (Eq.3) | 6 |
|  |  |  |  |

Table S4: The biochemical reaction rates *v^reaction^_n_ (x,t)* used in equation 4 and their dependency on concentration parameters *c_n_(x,t)* as obtained from the reaction network in Supplementary Table 1. Mass action kinetics apply for all reactions.

| ***v^reaction^_n_* Reaction** **term (Eq.4)** **and Formula** | |
| --- | --- |
| *v^reaction^_1_* = | 0.00047-0.0039*c1-6*c2*c1-8*6*c4*c1-2.4*c1*c3 |
| *v^reaction^_2_* = | -12*c2*c3 - 156*c2*c5+0.144*c7+420*c7*c16*c16-156*c17*c2+0.33*c14*c16-156*c20*c2-0.0058*c2+log(2)/2.3*c22 |
| *v^reaction^_3_* = | 6*c2*c1+8*6*c4*c1+2.4*c1*c3-156*c3*c5+0.144*c6-156*c3*c9+0.144*c10-156*c3*c11+0.144*c13+420*c6*c16*c16-156*c17*c3+4.45*c13*c16-156*c19*c3-0.0058*c3+420*c6*c16*c16-156*c17*c3+4.45*c13*c16-156*c19*c3-0.0058*c3 |
| *v^reaction^_4_* = | 12*c2*c3+12*c3*c7+12*c3*c14-0.0058*c4 |
| *v^reaction^_5_* = | 0.00073-0.0116*c5-156*c2*c5+0.144*c7-156*c3*c5+0.144*c6-12*c3*c5-420*c5*c16*c16+0.133*c17 |
| *v^reaction^_6_* = | 156*c3*c5-0.144*c6-12*c3*c6-420*c6*c16*c16+156*c17*c3-0.0347*c6 |
| *v^reaction^_7_* = | 156*c2*c5-0.144*c7-12*c3*c7-12*c3*c7-420*c7*c16*c16+156*c17*c2-0.0347*c7 |
| *v^reaction^_8_* = | 0 (not used, legacy parameter, kept in for further extensions) |
| *v^reaction^_9_* = | - 156*c3*c9+0.144*c10-12*c3*c9+12*c3*c7-420*c9*c16*c16+156*c18- 0.0058*c9 |
| *v^reaction^_10_* = | 156*c3*c9-0.144*c10-12*c3*c10-0.0058*c10 |
| *v^reaction^_11_* = | - 156*c3*c11+0.144*c13+12*c3*c5+12*c3*c7+12*c3*c9-4.45*c11*c16+31.9*c19-0.0058*c11 |
| *v^reaction^_12_*= | 12*c3*c5+12*c3*c6-0.33*c12*c16+14.2*c20-0.0347*c12 |
| *v^reaction^_13_* = | 156*c3*c11-0.144*c13+12*c3*c6+12*c3*c10-4.45*c13*c16+156*c19*c3-0.0058*c13 |
| *v^reaction^_14_* = | 12*c3*c7-12*c3*c14-0.33*c14*c16+156*c20*c2-0.0058*c14 |
| *v^reaction^_15_* = | 12*c3*c9+12*c3*c10+12*c3*c14-0.0347*c15 |
| *v^reaction^_16_* = | -2*(420*c5*c16*c16-0.133*c17 + 420*c7*c16*c16-156*c17*c2+420*c6*c16*c16-156*c17*c3)-4.45*c11*c16+31.9*c19-0.33*c12*c16+14.2*c20-4.45*c13*c16+156*c19*c3-0.33*c14*c16+156*c20*c2-2*(420*c9*c16*c16-156*c18)-0.0058*c16+log(2)/7*c23 |
| *v^reaction^_17_* = | -12*c3*c17+420*c5*c16*c16-0.133*c17+420*c7*c16*c16-156*c17*c2+420*c6*c16*c16-156*c17*c3-0.0347*c17 |
| *v^reaction^_18_* = | 420*c9*c16*c16-156*c18-0.0347*c18 |
| *v^reaction^_19_* = | 12*c3*c17+4.45*c11*c16-31.9*c19+4.45*c13*c16-156*c19*c3-0.0058*c19 |
| *v^reaction^_20_* = | 12*c3*c17+0.33*c12*c16-14.2*c20+0.33*c14*c16-156*c20*c2-0.0347*c20 |
| *v^reaction^_21_* = | -12*c21*c3 |
| *v^reaction^_22_* = | - log(2) / 2 . 3*c22 (starting at t _MOMP_ (x) ) |

**References**

1. Rehm M, Huber HJ, Dussmann H, Prehn JH: **Systems analysis of effector caspase activation and its control by X-linked inhibitor of apoptosis protein**. *Embo J* 2006, **25**(18):4338-4349.

2. Rehm M, Dussmann H, Prehn JH: **Real-time single cell analysis of Smac/DIABLO release during apoptosis**. *J Cell Biol* 2003, **162**(6):1031-1043.

3. Garcia-Calvo M, Peterson EP, Leiting B, Ruel R, Nicholson DW, Thornberry NA: **Inhibition of human caspases by peptide-based and macromolecular inhibitors**. *J Biol Chem* 1998, **273**(49):32608-32613.

4. Stennicke HR, Renatus M, Meldal M, Salvesen GS: **Internally quenched fluorescent peptide substrates disclose the subsite preferences of human caspases 1, 3, 6, 7 and 8**. *Biochem J* 2000, **350 Pt 2**:563-568.

5. Zou H, Yang R, Hao J, Wang J, Sun C, Fesik SW, Wu JC, Tomaselli KJ, Armstrong RC: **Regulation of the Apaf-1/caspase-9 apoptosome by caspase-3 and XIAP**. *J Biol Chem* 2003, **278**(10):8091-8098.

6. Riedl SJ, Renatus M, Schwarzenbacher R, Zhou Q, Sun C, Fesik SW, Liddington RC, Salvesen GS: **Structural basis for the inhibition of caspase-3 by XIAP**. *Cell* 2001, **104**(5):791-800.

7. Huang Y, Rich RL, Myszka DG, Wu H: **Requirement of both the second and third BIR domains for the relief of X-linked inhibitor of apoptosis protein (XIAP)-mediated caspase inhibition by Smac**. *J Biol Chem* 2003, **278**(49):49517-49522.

8. Eissing T, Conzelmann H, Gilles ED, Allgower F, Bullinger E, Scheurich P: **Bistability analyses of a caspase activation model for receptor-induced apoptosis**. *J Biol Chem* 2004, **279**(35):36892-36897.

9. Yoo SJ, Huh JR, Muro I, Yu H, Wang L, Wang SL, Feldman RM, Clem RJ, Muller HA, Hay BA: **Hid, Rpr and Grim negatively regulate DIAP1 levels through distinct mechanisms**. *Nat Cell Biol* 2002, **4**(6):416-424.

10. Cain K, Brown DG, Langlais C, Cohen GM: **Caspase activation involves the formation of the aposome, a large (approximately 700 kDa) caspase-activating complex**. *J Biol Chem* 1999, **274**(32):22686-22692.
