# Supplementary material for: Diffusion is capable of translating anisotropic apoptosis initiation into a homogeneous execution of cell death
Source: BMC Syst Biol. 2010 Feb 4;4:9. doi: 10.1186/1752-0509-4-9 (PMC2831829; doi:10.1186/1752-0509-4-9)
Supplement: Additional file 2 — Comparison of diffusive signal spread between simplified one and three dimensional scenarios. Comparison of signal spread between simplified one and a three dimensional spatial models. The one dimensional model represents a linear slab with the input pulse located at the left boundary (A). The three dimensional model represents a sphere with the input signal starting synchronously on the entire surface of the sphere (B). Signal progression along the slab (1 dimensional model) or towards the centre of the sphere (3 dimensional model) was investigated. To mathematically handle the 3-dimensional spherical model with the PDEPE subroutine in MATLAB, the diffusion process was transformed to a problem of one spatial and temporal component without loss of information. This yielded the following reaction diffusion equation for the radial component of species n: where r is the radius and Dn denotes the diffusion coefficient. Only small discrepancies were observed between the two scenarios. More complex scenarios such as spatially anisotropic triggers could not be subjected to this dimension reduction. [file 1752-0509-4-9-S2.PDF]

Additional File 2

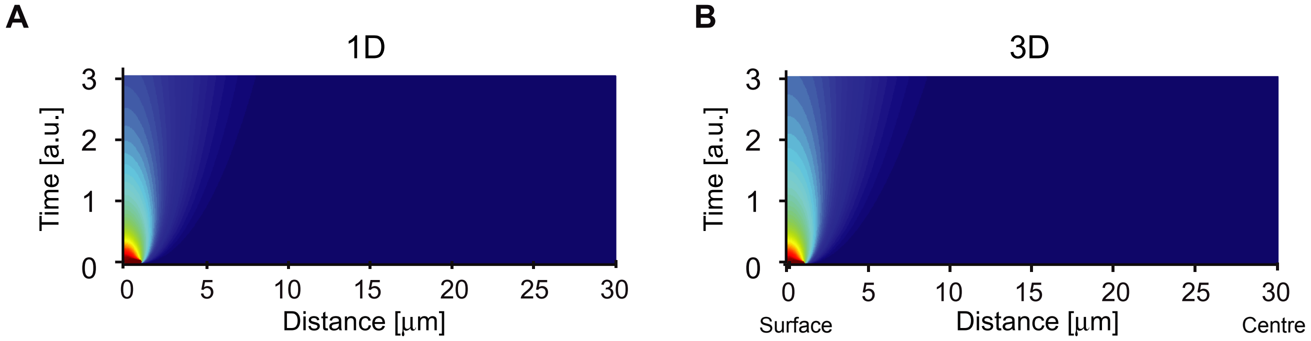

Figure Legend Additional File 2: Comparison of diffusive signal spread between simplified one and three dimensional scenarios.

Comparison of signal spread between simplified one and a three dimensional spatial models. The one dimensional model represents a linear slab with the input pulse located at the left boundary (A). The three dimensional model represents a sphere with the input signal starting synchronously on the entire surface of the sphere (B). Signal progression along the slab (1 dimensional model) or towards the centre of the sphere (3 dimensional model) was investigated. Color code represents decreasing concentrations from red to deep blue.

To mathematically handle the 3-dimensional spherical model with the PDEPE subroutine in MATLAB, the diffusion process was transformed to a problem of one spatial and temporal component without loss of information. This yielded the following reaction diffusion equation for the radial component $c^{rad}{}_n$ of species n:

$$\partial c^{rad}{}_n(r,t) / \partial t = D_n \frac{1}{r^2} \partial / \partial r \left(r^2 \, \partial c^{rad}{}_n(r,t) / \partial r \right)$$

where r is the radius and D_n denotes the diffusion coefficient. Only small discrepancies were observed between the two scenarios. More complex scenarios such as spatially anisotropic triggers could not be subjected to this dimension reduction.
