# Supplementary material for: Diffusion is capable of translating anisotropic apoptosis initiation into a homogeneous execution of cell death
Source: BMC Syst Biol. 2010 Feb 4;4:9. doi: 10.1186/1752-0509-4-9 (PMC2831829; doi:10.1186/1752-0509-4-9)
Supplement: Additional file 4 — Spatially homogeneous caspase activation in HeLa cervical cancer cells. Substrate cleavage by effector caspases was experimentally measured by CFP-DEVD-YFP FRET disruption at fast sampling rates in HeLa cells. FRET disruption was measured in regions at distal ends of the cell. The traces shown were obtained from a cell treated with 100 ng/ml TRAIL/1 μg/ml CHX. Arrow indicates onset of substrate cleavage. Corresponding results were obtained from n = 19 additional cells treated with TRAIL/CHX and n = 14 cells treated with 1 μM STS. [file 1752-0509-4-9-S4.PDF]

Additional File 4

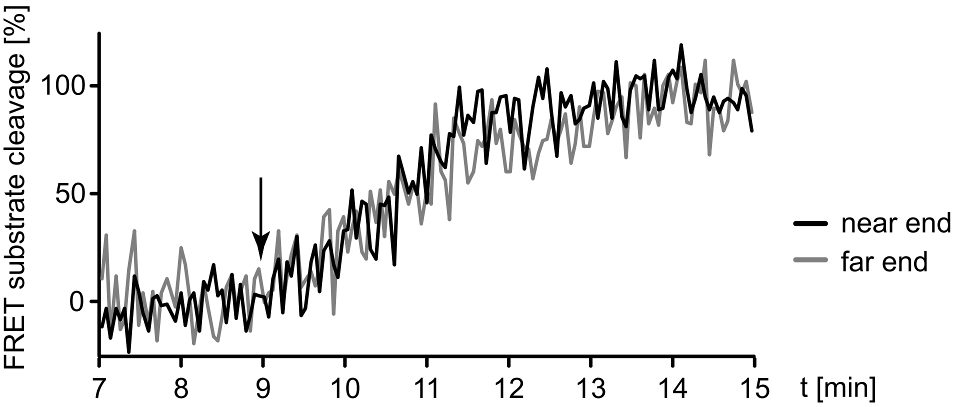

Figure Legend Additional File 4: Spatially homogeneous caspase activation in HeLa cervical cancer cells.

Substrate cleavage by effector caspases was experimentally measured by CFP-DEVD-YFP FRET disruption at fast sampling rates in HeLa cells. FRET disruption was measured in regions at distal ends of the cell. The traces shown were obtained from a cell treated with 100 ng/ml TRAIL/1 μg/ml CHX. Arrow indicates onset of substrate cleavage. Corresponding results were obtained from n = 19 additional cells treated with TRAIL/CHX and n = 14 cells treated with 1 μM STS.
