# Supplementary material for: Diffusion is capable of translating anisotropic apoptosis initiation into a homogeneous execution of cell death
Source: BMC Syst Biol. 2010 Feb 4;4:9. doi: 10.1186/1752-0509-4-9 (PMC2831829; doi:10.1186/1752-0509-4-9)
Supplement: Additional file 5 — MatLab script of the reaction-diffusion model. The file contains the MatLab code for the reaction-diffusion model and the required annotations to repeat all modelling presented in this study. The model could not be provided as SBML as spatiotemporal PDE models are not yet supported. [file 1752-0509-4-9-S5.HTML]

rdsimall


 


```
%%%%%%%%%%%%%%%%%%%%%%%%%%%%%%%%%%%%%%%%%%%%%%%%%%%%%%%%%%%%%%%%%%%%%%%%%%%%%%%%%%%%%%%
% Supplement to: Huber, Laussmann, Prehn and Rehm
%
% (C)   Heinrich Huber, 2008-2009
%       Royal College of Surgeons, Dublin
%
%       Spatiotemporal Systems Model of Apoptosis Execution
%
% Note:
%
% - The model consists of 53 reaction with 20 parameters (+substrate
%   cleavage and two species to generate the input function) derived from (Rehm et al EMBO-J 25, 4338-4349, 2006)
%
% - Indirect addressing is used to build up the reaction velocities (see description below)
%
% - Two input functions (cyt-c driven apoptosome formation and SMAC release) are modelled
%   by pseudo first order chemical reactions
%
% - Input function 1: Non-active APAF1 gets activated: Reaction 54
%   generates a funtion that leads to APAF1_active(t) =  APAF1_active_final*(1-exp(log(2)*t/t12_apaf))
%
% - Input function 2: Smac gets released from the mitochodria: Reaction 55
%   generates funtion that leads to SMAC_cytosol(t) =  SMAC_cytosol_final*(1-exp(log(2)*t/t12_SMAC))
%
% - Spatial delay: Input function starts at spatial point x at t = k*x (with velocity = 1/k= 0.1 um/s)
%   generates funtion that leads to SMAC_cytosol(t) =  SMAC_cytosol_final*(1-exp(log(2)*t/t12_SMAC))
%
%     Please email comments and suggestions to heinhuber@rcsi.ie
%%%%%%%%%%%%%%%%%%%%%%%%%%%%%%%%%%%%%%%%%%%%%%%%%%%%%%%%%%%%%%%%%%%%%%%%%%%%%%%%%%%%%%%%
%

function rdsimall


%   Parameters of the unperturbed network (Fig 2-4)

sub01       = 0.01;     % check for substrate 1%
sub50       = 0.5;      % check for substrate 50%
idiff       = 0;        % Apoptosome can diffuse according to Stokes-Einstein
x_enzyme    = 1;        % no change in biochemical reactivity
ifeedback   = 11;       % both feedback C3/C9 in operation
downfact    = 1;        % no downregulation of diffusion
%
%   calculate Fig 2 and Fig 3 B,D,F
%
IMODE =0;
[tdummy1,tdummy2]=rdsim (IMODE, downfact, idiff,x_enzyme,sub01,sub50,ifeedback);
%
%   calculate Fig 3 A,C,E
%
IMODE =1;
[tdummy1,tdummy2]=rdsim (IMODE, downfact, idiff,x_enzyme,sub01,sub50,ifeedback);
%
%   calculate Fig 4 C,D,E,F
%
IMODE =2;
downfact    = 10;        %Select downregulation factor
[tdummy1,tdummy2]=rdsim (IMODE, downfact, idiff,x_enzyme,sub01,sub50,ifeedback);
%
%   calculate Fig 4 B
%
IMODE =3;
[tdummy1,tdummy2]=rdsim (IMODE, downfact, idiff,x_enzyme,sub01,sub50,ifeedback);


%  calculate Fig 4A, Fig 5 & 6

IMODE       = 4;
%
Fig4A        = zeros(2,5);
Fig6A        = zeros(2,5);
Fig6B        = zeros(2,5);
Fig6C        = zeros(2,5);
Fig6D        = zeros(2,5);
Fig6E        = zeros(2,5);
Fig6F        = zeros(2,5);
Fig5A        = zeros(2,5);
Fig5C        = zeros(2,5);
%
%   Reference case Fig 4A (unperturbed)
%
x_enzyme    = 1;        % no change in biochemical reactivity
idiff       = 0;        % Apoptosome can diffuse according to Stokes-Einstein
ifeedback   = 11;       % both feedbacks (capase-9 and caspase-3) are intact
sub01       = 0.01;     % check for substrate 1%
sub50       = 0.5;      % check for substrate 50%
%
[t1,t2]=rdsim (IMODE, 1, idiff,x_enzyme,sub01,sub50,ifeedback);
Fig4A(1,1) = t1;
Fig4A(2,1) = t2;
[t1,t2]=rdsim (IMODE, 10, idiff,x_enzyme,sub01,sub50,ifeedback);
Fig4A(1,2) = t1;
Fig4A(2,2) = t2;
[t1,t2]=rdsim (IMODE, 100, idiff,x_enzyme,sub01,sub50,ifeedback);
Fig4A(1,3) = t1;
Fig4A(2,3) = t2;
[t1,t2]=rdsim (IMODE, 1000, idiff,x_enzyme,sub01,sub50,ifeedback);
Fig4A(1,4) = t1;
Fig4A(2,4) = t2;
[t1,t2]=rdsim (IMODE, 10000, idiff,x_enzyme,sub01,sub50,ifeedback);
Fig4A(1,5) = t1;
Fig4A(2,5) = t2;
%
%   Fig 5A: Perturbed Network
%   no change in biochemical reactivity, Apoptosome blocked, no
%   C9-feedback
%
x_enzyme    = 1;            % no change in biochemical reactivity
idiff       = 0;            % Apoptosome can diffuse according to Stokes-Einstein
ifeedback   = 10;           %  no C9-feedback, C3 auto-activation intact
%
[t1,t2]=rdsim (IMODE, 1, idiff,x_enzyme,sub01,sub50,ifeedback);
Fig5A(1,1) = t1;
Fig5A(2,1) = t2;
[t1,t2]=rdsim (IMODE, 10, idiff,x_enzyme,sub01,sub50,ifeedback);
Fig5A(1,2) = t1;
Fig5A(2,2) = t2;
[t1,t2]=rdsim (IMODE, 100, idiff,x_enzyme,sub01,sub50,ifeedback);
Fig5A(1,3) = t1;
Fig5A(2,3) = t2;
[t1,t2]=rdsim (IMODE, 1000, idiff,x_enzyme,sub01,sub50,ifeedback);
Fig5A(1,4) = t1;
Fig5A(2,4) = t2;
[t1,t2]=rdsim (IMODE, 10000, idiff,x_enzyme,sub01,sub50,ifeedback);
Fig5A(1,5) = t1;
Fig5A(2,5) = t2;
%
%   Fig 5C:  Perturbed Network
%   no acceleration of enzyme activity, Apoptosome blocked, no C3-feedback
%
x_enzyme    = 1;            % no change in biochemical reactivity
idiff       = 0;            % Apoptosome can diffuse according to Stokes-Einstein
ifeedback   = 01;           % C9-feedback intact, C3 auto-activation intact
%
[t1,t2]=rdsim (IMODE, 1, idiff,x_enzyme,sub01,sub50,ifeedback);
Fig5C(1,1) = t1;
Fig5C(2,1) = t2;
[t1,t2]=rdsim (IMODE, 10, idiff,x_enzyme,sub01,sub50,ifeedback);
Fig5C(1,2) = t1;
Fig5C(2,2) = t2;
[t1,t2]=rdsim (IMODE, 100, idiff,x_enzyme,sub01,sub50,ifeedback);
Fig5C(1,3) = t1;
Fig5C(2,3) = t2;
[t1,t2]=rdsim (IMODE, 1000, idiff,x_enzyme,sub01,sub50,ifeedback);
Fig5C(1,4) = t1;
Fig5C(2,4) = t2;
[t1,t2]=rdsim (IMODE, 10000, idiff,x_enzyme,sub01,sub50,ifeedback);
Fig5C(1,5) = t1;
Fig5C(2,5) = t2;%
%
%   Fig 6A:  Perturbed Network
%   10 times increased activity, Apoptosome can diffuse, feedback intact
%
x_enzyme    = 10;       % 10 times increased activity
idiff       = 0;        % Apoptosome can diffuse according to Stokes-Einstein
ifeedback   = 11;       % both feedbacks (capase-9 and caspase-3 auto-activation) are intact
%
[t1,t2]=rdsim (IMODE, 1, idiff,x_enzyme,sub01,sub50,ifeedback);
Fig6A(1,1) = t1;
Fig6A(2,1) = t2;
[t1,t2]=rdsim (IMODE, 10, idiff,x_enzyme,sub01,sub50,ifeedback);
Fig6A(1,2) = t1;
Fig6A(2,2) = t2;
[t1,t2]=rdsim (IMODE, 100, idiff,x_enzyme,sub01,sub50,ifeedback);
Fig6A(1,3) = t1;
Fig6A(2,3) = t2;
[t1,t2]=rdsim (IMODE, 1000, idiff,x_enzyme,sub01,sub50,ifeedback);
Fig6A(1,4) = t1;
Fig6A(2,4) = t2;
[t1,t2]=rdsim (IMODE, 10000, idiff,x_enzyme,sub01,sub50,ifeedback);
Fig6A(1,5) = t1;
Fig6A(2,5) = t2;
%
%   Fig 6C:  Perturbed Network
%   no acceleration of enzyme activity, Apoptosome blocked, feedback intact
%
x_enzyme    = 1;        % no acceleration of enzyme activity
idiff       = 1;        % Apoptosome blocked
ifeedback   = 11;       % both feedbacks (capase-9 and caspase-3) are intact
%
[t1,t2]=rdsim (IMODE, 1, idiff,x_enzyme,sub01,sub50,ifeedback);
Fig6C(1,1) = t1;
Fig6C(2,1) = t2;
[t1,t2]=rdsim (IMODE, 10, idiff,x_enzyme,sub01,sub50,ifeedback);
Fig6C(1,2) = t1;
Fig6C(2,2) = t2;
[t1,t2]=rdsim (IMODE, 100, idiff,x_enzyme,sub01,sub50,ifeedback);
Fig6C(1,3) = t1;
Fig6C(2,3) = t2;
[t1,t2]=rdsim (IMODE, 1000, idiff,x_enzyme,sub01,sub50,ifeedback);
Fig6C(1,4) = t1;
Fig6C(2,4) = t2;
[t1,t2]=rdsim (IMODE, 10000, idiff,x_enzyme,sub01,sub50,ifeedback);
Fig6C(1,5) = t1;
Fig6C(2,5) = t2;
%
%   Fig 6E:  Perturbed Network
%   10 times enzyme speed, Apoptosome blocked, feedback intact
%
x_enzyme    = 10;       % no acceleration of enzyme activity
idiff       = 1;        % Apoptosome blocked
ifeedback   = 11;       % both feedbacks (capase-9 and caspase-3) are intact
%
[t1,t2]=rdsim (IMODE, 1, idiff,x_enzyme,sub01,sub50,ifeedback);
Fig6E(1,1) = t1;
Fig6E(2,1) = t2;
[t1,t2]=rdsim (IMODE, 10, idiff,x_enzyme,sub01,sub50,ifeedback);
Fig6E(1,2) = t1;
Fig6E(2,2) = t2;
[t1,t2]=rdsim (IMODE, 100, idiff,x_enzyme,sub01,sub50,ifeedback);
Fig6E(1,3) = t1;
Fig6E(2,3) = t2;
[t1,t2]=rdsim (IMODE, 1000, idiff,x_enzyme,sub01,sub50,ifeedback);
Fig6E(1,4) = t1;
Fig6E(2,4) = t2;
[t1,t2]=rdsim (IMODE, 10000, idiff,x_enzyme,sub01,sub50,ifeedback);
Fig6E(1,5) = t1;
Fig6E(2,5) = t2;

%
%   Difference plots
%
Fig6B = Fig6A - Fig4A;
Fig6D = Fig6C - Fig4A;
Fig6F = Fig6E - Fig4A;
Fig5B = Fig5A - Fig4A;
Fig5D = Fig5C - Fig4A;
save('testdata','Fig4A','Fig6A','Fig6B','Fig6C','Fig6D','Fig6E','Fig6F','Fig5A','Fig5B','Fig5C','Fig5D')
%
%   Plot Figure 4A
%
figure
subplot(3,2,1), bar(Fig4A')
set(gca,'FontSize',8)
set(gca,'FontName','Arial')
set (gca, 'LineWidth',2)
xlabel('Time after model initiation [min]')
ylabel('Fold reduction in diffusivity')
set (gca, 'LineWidth',2)
%
%   Plot Figure 5
%
figure
subplot(3,2,1), bar(Fig5A')
set(gca,'FontSize',8)
set(gca,'FontName','Arial')
set (gca, 'LineWidth',2)
xlabel('Time after model initiation [min]')
ylabel('Fold reduction in diffusivity')
set (gca, 'LineWidth',2)
%
subplot(3,2,2), bar(Fig5B')
set(gca,'FontSize',8)
set(gca,'FontName','Arial')
set (gca, 'LineWidth',2)
xlabel('Time after model initiation [min]')
ylabel('Fold reduction in diffusivity')
set (gca, 'LineWidth',2)
%
subplot(3,2,3), bar(Fig5C')
set(gca,'FontSize',8)
set(gca,'FontName','Arial')
set (gca, 'LineWidth',2)
xlabel('Time after model initiation [min]')
ylabel('Fold reduction in diffusivity')
set (gca, 'LineWidth',2)
%
subplot(3,2,4), bar(Fig5D')
set(gca,'FontSize',8)
set(gca,'FontName','Arial')
set (gca, 'LineWidth',2)
xlabel('Time after model initiation [min]')
ylabel('Fold reduction in diffusivity')
set (gca, 'LineWidth',2)
%
%   Plot Figure 6
%
figure
subplot(3,2,1), bar(Fig6A')
set(gca,'FontSize',8)
set(gca,'FontName','Arial')
set (gca, 'LineWidth',2)
xlabel('Time after model initiation [min]')
ylabel('Fold reduction in diffusivity')
set (gca, 'LineWidth',2)
%
subplot(3,2,2), bar(Fig6B')
set(gca,'FontSize',8)
set(gca,'FontName','Arial')
set (gca, 'LineWidth',2)
xlabel('Time after model initiation [min]')
ylabel('Fold reduction in diffusivity')
set (gca, 'LineWidth',2)
%
subplot(3,2,3), bar(Fig6C')
set(gca,'FontSize',8)
set(gca,'FontName','Arial')
set (gca, 'LineWidth',2)
xlabel('Time after model initiation [min]')
ylabel('Fold reduction in diffusivity')
set (gca, 'LineWidth',2)
%
subplot(3,2,4), bar(Fig6D')
set(gca,'FontSize',8)
set(gca,'FontName','Arial')
set (gca, 'LineWidth',2)
xlabel('Time after model initiation [min]')
ylabel('Fold reduction in diffusivity')
set (gca, 'LineWidth',2)
%
subplot(3,2,5), bar(Fig6E')
set(gca,'FontSize',8)
set(gca,'FontName','Arial')
set (gca, 'LineWidth',2)
xlabel('Time after model initiation [min]')
ylabel('Fold reduction in diffusivity')
set (gca, 'LineWidth',2)
%
subplot(3,2,6), bar(Fig6F')
set(gca,'FontSize',8)
set(gca,'FontName','Arial')
set (gca, 'LineWidth',2)
xlabel('Time after model initiation [min]')
ylabel('Fold reduction in diffusivity')
set (gca, 'LineWidth',2)


end


function [it1,it2]= rdsim (IMODE, downfact, idiff,x_enzyme,stest1,stest2,ifeed)
global km kp S vel sbreakdown npartners nreactions tmax ix0 k_space
global ksub tmax xnorm xcol xo diff xmax

%%%%%%%%%%%%%%%%%%%%%%%%%%%%%%%%%%%%%%%%%%%%%%%%%%%%%%%%%%%%%%%%%%%%%%%%%%%%%%%%%%%%%%%%
%
% - IMODE =0, No Diffusion, Fig. 2, Fig 3. B,D,F
%
% - IMODE =1, Free diffusion, Fig. 3 A,C,E
%
% - IMODE =2, Blocked Diffusion /Impaired Diffusivity Fig. 4 C-F
%
% - IMODE =3, Diffusion sensitivity analysis, Fig. 4 B
%
% - downfact, Impairment factor for diffusion (for IMODE =2,4)
%
%   The following parameters are only used for the regulation studies Fig. 5, 6
%
% - idiff =0, Apoptosome dependent entities diffuse according to Stokes-Einstein
%
% - idiff =1, Apoptosome dependent entities blocked
%
% - x_enzyme, acceleration factor of all enzyme reactions (equilibrium unchanged)
%
% - stest1,stest2, values of substrates that are tested for time of cleavage
%
% - ifeed = 11, Caspase-3 and caspase-9 feedback in operation
%
% - ifeed = 01, Caspase-3 feedback blocked, caspase-9 in operation
%
% - ifeed = 10, Caspase-3 feedback in operation, caspase-9 blocked
%
% - ifeed = 00, both feedbacks blocked
%
%
%     Please email comments and suggestions to heinhuber@rcsi.ie
%%%%%%%%%%%%%%%%%%%%%%%%%%%%%%%%%%%%%%%%%%%%%%%%%%%%%%%%%%%%%%%%%%%%%%%%%%%%%%%%%%%%%%%%

it1 = 0;        % Dummy initialization
it2 = 0;        % Dummy initialization

%
% assign calculation parameter
%
switch IMODE
    %
    %       Set calculation mode to obtain the desired figues
    %
    case 0
        pagfpd  = 0;                 % no diffusion
        hx      = 0.25;              % x-spacing (fine)

    case 1
        pagfpd  = 24*60;             % Reference diffusion rate of PAGFP in uM**2 * min**(-1)
        hx      = 0.25;              % x-spacing (fine)

    case 2
        pagfpd  = 24*60/downfact;   % Plots for substrate cleavage with downregulated diffusion rate
                                    % Fig 6 c-f
        hx      = 0.25;             % x-spacing (fine)

    case 3
        pagfpd  = 24*60;            % Reference diffusion rate of PAGFP in uM**2 * min**(-1)
        hx      = 1;                % x-spacing

    case 4
        pagfpd  = 24*60/downfact;   % Plots for substrate cleavage with downregulated diffusion rate
                                    % Fig 6 c-f
        hx      = 0.25;             % x-spacing (fine)


        % Regulation studies of diffusion coefficients as of Fig 6 b
end

%
% Biochemical constants from Rehm 2006
%

XIAP            = 0.063;            % initial XIAP concentration
PCAS3           = 0.120;            % initial Procaspase-3 concentration
a0              = 3.325;            % initial APAF-1 concentration
casp9           = 0.03;             % initial Procaspase-9 concentration
smac0           = 0.125;            % initial mitochondrial Smac concentration
sbreakdown      = 0.00001;          % Protein production breaks down for 1 %
tmax            = 25;               % max time for reaction model
dt              = 0.1;              % time step for output
t12             = 2.3;              % Half time of Apoptosome formation (see Rehm 2006)
t12smac         = 7;                % Half time of Apoptosome formation (see Rehm 2003)
%
%   take lowest of Caspase 9 or Apaf1 for apoptosome formation (restricting value)
%
if casp9 < a0
    a0 = casp9;
end
%
%   cell size and input signal propagation
%
xmax            = 30;               % 30 um cell length (1D)
t12propagation  = (xmax/ 0.1)/60;   % End to End propagation time (velocity 0.1 um/s)

%%%%%%%%%%%%%%%%%%%%%%%%%%%%%%%%%%%%%%%%%%%%%%%%%%%%%%%%%%%%%%%%%%%%%%%%%%%%%%%%%%%%%%%
% Conventions / Fractions
%%%%%%%%%%%%%%%%%%%%%%%%%%%%%%%%%%%%%%%%%%%%%%%%%%%%%%%%%%%%%%%%%%%%%%%%%%%%%%%%%%%%%%%%

%1	C3_                     : Procaspase 3
%2	C9a                     : Caspase 9(p35/p12) bound to the apoptosome
%3	C3a                     : free active Caspase-3
%4	C9H                     : Caspase 9 bound to the apoptosome, p35/p10
%5	XIAP                    : free XIAP
%6	[XIAP~C3a]              : XIAP bound to Caspase 3
%7	[XIAP~C9a]              : XIAP bound to Caspase 9 (p35/p12)
%8	[XIAP~C9a~C3a]          : XIAP bound to Caspase 9 (p35/p12) and Caspase 3
%9	[XIAP~{cl-C9a}]         : XIAP bound to the p2-fragment
%10	[[XIAP~{cl-C9a}]~C3a]   : XIAP bound to the p2-fragment and active Caspase 3
%11	BIR12                   : XIAP fragment of domains BIR1 and BIR2
%12	BIR3R                   : XIAP fragment of domains BIR3 and RING
%13	[BIR12~C3a]             : BIR12 bound to Caspase 3
%14	[BIR3R~C9a]             : BIR3R bound to Caspase 9 (p35/p12)
%15	[BIR3R~{cl-C9a}]        : BIR3R bound to the p2-fragment
%16	SMAC                    : free SMAC
%17	[XIAP~2SMAC]            : XIAP bound to 2SMAC
%18	[[XIAP~{cl-C9a}]~  SMAC]: XIAP bound to the p2-fragment and SMAC
%19	[BIR12~SMAC]            : BIR12 bound to 2SMAC
%20	[BIR3R~SMAC]            : BIR3R bound to SMAC
%21 Substrate               : FRET substrate
%22 APAF1                   : Free APAF1
%23 SMACmito                : 'Mitochondrial' Smac
%24 DUMMY1                  : Dummy value for calculation, always = 1


%%%%%%%%%%%%%%%%%%%%%%%%%%%%%%%%%%%%%%%%%%%%%%%%%%%%%%%%%%%%%%%%%%%%%%%%%%%%%%%%%%%%%%%
% Basic Constants and Definitions
%%%%%%%%%%%%%%%%%%%%%%%%%%%%%%%%%%%%%%%%%%%%%%%%%%%%%%%%%%%%%%%%%%%%%%%%%%%%%%%%%%%%%%%%

% Flags
% Protein turnover stops after 90 % substrate cleavage
% > 100 no production

xcol        = zeros(2,200);
%
%start values
%
kp          =  zeros(74,1);        % kinetic constants for forward reactions
km          =  zeros(74,1);        % kinetic constants for backward reactions
npartners   = 23;                  % Number of partners
nreactions  = 55;                  % Number of reactions
mass        = zeros(npartners,1);  % pre-allocation for vector of protein masses
diff        = zeros(npartners,1);  % pre-allocation for vector of diffusion constants

% mesh parameters

xmin        = 0;
t0          = 0;                    % initial time
tf          = tmax;                 % final time
ht          = 0.01;                % t-spacing
xlist       = xmin:hx:xmax;         % Spatial discretization
tlist       = t0:ht:tf;             % Temporal discretization


if ifeed ==00
   xfeedc3 = 0;
   xfeedc9c3 = 0;
%   'no  feedback at all'
   tmax = 5*tmax;
   tf          = tmax;                 % final time

elseif ifeed ==01
   xfeedc3 = 0;
   xfeedc9c3 = 1;
%    'no C3 feedback'

elseif ifeed ==10
   xfeedc3 = 1;
   xfeedc9c3 = 0;
%    'no C9 feedback'
   tmax = 5*tmax;
   tf          = tmax;                 % final time

elseif ifeed ==11
   xfeedc3 = 1;
   xfeedc9c3 = 1;
%    'both feedbacks'
end

%%%%%%%%%%%%%%%%%%%%%%%%%%%%%%%%%%%%%%%%%%%%%%%%%%%%%%%%%%%%%%%%%%%%%%%%%%%%%%%%%%%%%%%
% Kinetic parameter (Standard)
%%%%%%%%%%%%%%%%%%%%%%%%%%%%%%%%%%%%%%%%%%%%%%%%%%%%%%%%%%%%%%%%%%%%%%%%%%%%%%%%%%%%%%%%

ksub = 12;              %substrate cleavage rate = C3-activity from Stennicke 2000

% Forward Parameter
                        % kp( 1) will be calculated (normed to C3-concentration)
                        % kp( 2) will be calculated (normed to XIAP concentration)
kp( 3) = 6 ;            %Garcia-Calvo 1998
kp( 4) = xfeedc9c3*12;  %Caspase 3 activity from Stennicke 2000
kp( 5) = 8*kp(3);       %8-fold feedback Zuo2002
kp( 6) = xfeedc3*2.4;   %Autofeedback 5 times lower that activity Zuo2002
kp( 7) = 156;           %Caspase-3-XIAP inhibition Riedl2001
kp( 8) = 0;             %Caspase-3-XIAP inhibition (with C9 binding) Riedl2001
kp( 9) = 0;             %Caspase-3-XIAP inhibition (with p2 binding) Riedl2001
kp(10) = 156;           %BIR12-XIAP binding taken from C3, Riedl2001
kp(11) = 12;            %Caspase 3 activity from Stennicke 2000
kp(12) = 12;            %Caspase 3 activity from Stennicke 2000
kp(13) = 12;            %Caspase 3 activity from Stennicke 2000
kp(14) = 12;            %Caspase 3 activity from Stennicke 2000
kp(15) = 12;            %Caspase 3 activity from Stennicke 2000
kp(16) = 12;            %Caspase 3 activity from Stennicke 2000
kp(17) = 12;            %Caspase 3 activity from Stennicke 2000
kp(18) = 12;            %Caspase 3 activity from Stennicke 2000
kp(19) = 12;            %Caspase 3 activity from Stennicke 2000
kp(20) = 12;            %Caspase 3 activity from Stennicke 2000
kp(21) = 156;           %Caspase 9-XIAP clustering assumed as Caspase3  Riedl2001
kp(22) = 0;             %Caspase 9-XIAP clustering assumed as Caspase3  Riedl2001
kp(23) = 156;           %BIR3R-XIAP  clustering assumed as Caspase3  Riedl2001
kp(24) = 0;             %no decay of complex cleaved from the apoptosome, reaction neglected
kp(25) = 0;             %no decay of complex cleaved from the apoptosome, reaction neglected
kp(26) = 420;           %Smac-XIAP binding from Huang2003
kp(27) = 420;           %Smac-XIAP with concurrence to C9a as plain-Smac-XIAP binding from Huang2003
kp(28) = 420;           %Smac-XIAP with concurrence to C3a as plain-Smac-XIAP binding from Huang2003
kp(29) = 0;             %Smac-XIAP with concurrence to C3a&C9a as plain-Smac-XIAP binding from Huang2003
kp(30) = 4.45;          %Smac-BIR12 from Huang2003
kp(31) = 0.33;          %Smac-BIR3R from Huang2003
kp(32) = 4.45;          %Smac-BIR12 with concurrence to C3a as plain-Smac-BIR12 binding from Huang2003
kp(33) = 0.33;          %Smac-BIR3R with concurrence to C3a as plain-Smac-BIR3R binding from Huang2003
kp(34) = 420;           %Smac-clustering to the XIAP-p2 fragment as plain-Smac-XIAP binding from Huang2003
kp(35) = 0.0058;        %C9H normal degradation taken from Eissing2004 (120min)
kp(36) = 0.0058;        %C9A normal degradation taken from Eissing2004 (120min)
kp(37) = 0.0058;        %C3A normal degradation taken from Eissing2004 (120min)
kp(38) = 0.0347;        %[XIAP~C3a] enforced degradation taken from Yoo2002 (20 min)
kp(39) = 0.0347;        %[XIAP~C3a~C9a]  enforced degradation taken from Yoo2002 (20 min)
kp(40) = 0.0347;        %[XIAP~C9a] enforced degradation taken from Yoo2002 (20 min)20
kp(41) = 0.0058;        %[XIAP~C9a] no enforced degradation of apoptosomal caspase 9 taken from Eissing2004 (120min)
kp(42) = 0.0347;        %[XIAP~{cl-C9a}] enforced degradation of non apoptosomal caspase 9 taken from Yoo2002 (20 min)
kp(43) = 0.0347;        %[XIAP~{cl-C9a}]~  SMAC]enforced degradation taken from Yoo2002 (20 min)
kp(44) = 0.0347;        %[XIAP~2SMAC]  enforced degradation taken from Yoo2002 (20 min)
kp(45) = 0.0058;        %BIR12_normal degradation taken from Eissing2004 (120min)
kp(46) = 0.0347;        %BIR3R_enforced degradation taken from Yoo2002 (20 min)
kp(47) = 0.0058;        %[BIR12~SMAC]normal degradation taken from Eissing2004 (120min)
kp(48) = 0.0347;        %[BIR3R~SMAC] enforced degradation taken from Yoo2002 (20 min)
kp(49) = 0.0058;        %[BIR12~C3a]normal degradation taken from Eissing2004 (120min)
kp(50) = 0.0058;        %[BIR3R~C9a] enforced degradation of non apoptosomal caspase 9 (20 min)
kp(51) = 0.0347;        %[BIR3R~{cl-C9a}] enforced degradation of non apoptosomal caspase 9 taken from Yoo2002 (20 min)
kp(52) = 0.0058;        %SMAC_ normal degradation taken from Eissing2004 (120min)
kp(53) = ksub;          %substrate cleavage, Stennicke 2000
%
%   The following reactions are the input functions
%
%   inactive APAF1      -> gets converted to active APAF1 ("Apoptosome formation")
%   mitochondrial SMAC  -> cytosolic SMAC (SMAC release)
%
kp(54) =  log(2)/t12;           %Apoptosome formation kinetics Rehm 2006
kp(55) =  log(2)/t12smac;       %SMAC release Rehm 2003
%

% Backward Parameter (0 for irreversible reactions)

km( 1) = 0.0039;    %relaxation part of the C3 turnover rate taken from Eissing2004
km( 2) = 0.0116;    %relaxation part of the XIAP turnover rate taken from Eissing2004
km( 3) = 0;
km( 4) = 0;
km( 5) = 0;
km( 6) = 0;
km( 7) = 0.144;     % Caspase-3-XIAP inhibition Riedl2001
km( 8) = 0.;
km( 9) = 0.;
km(10) = 0.144;     % BIR12-XIAP binding taken from C3, Riedl2001
km(11) = 0;
km(12) = 0;
km(13) = 0;
km(14) = 0;
km(15) = 0;
km(16) = 0;
km(17) = 0;
km(18) = 0;
km(19) = 0;
km(20) = 0;
km(21) = 0.144;     % Caspase 9-XIAP clustering assumed as Caspase3  Riedl2001
km(22) = 0;
km(23) = 0.144;     % BIR3R-XIAP  clustering assumed as Caspase3  Riedl2001
km(24) = 0;
km(25) = 0;
km(26) = 0.133;	    %Smac-XIAP binding from Huang2003
km(27) = 156;	    %from Kon for XIAP-C3 clustering Riedl2001 (for concurrence reaction setting C3 free)
km(28) = 156;	    %from Kon for XIAP-C9 clustering Riedl2001 (for concurrence reaction setting C9 free)
km(29) = 0;
km(30) = 31.9;	    %Smac-BIR12 from Huang2003
km(31) = 14.2;	    %Smac-BIR3R from Huang2003
km(32) = 156;	    %from Kon for XIAP-C3 clustering Riedl2001 (for concurrence reaction setting C3 free)
km(33) = 156;	    %from Kon for XIAP-C9 clustering Riedl2001 (for concurrence reaction setting C9 free)
km(34) = 156;       %from Kon for XIAP-C9-C3 clustering Riedl2001 (for concurrence reaction setting C9,C3 free)
km(35:55) = 0;

% Mass parameter [kD]

mass( 1) = 32;      %Procaspase 3
mass( 2) = 700;     %Caspase 9(p35/p12) bound to the apoptosome
mass( 3) = 46;      %free active Caspase-3
mass( 4) = 686;     %Caspase 9 bound to the apoptosome, p35/p10
mass( 5) = 57;      %free XIAP
mass( 6) = 87;      %XIAP bound to Caspase 3
mass( 7) = 757;     %XIAP bound to Caspase 9 (p35/p12) bound to the apoptosome
mass( 8) = 760;     %XIAP bound to Caspase 9 (p35/p12) and Caspase 3, bound to the apoptosome , legacy parameter, not used
mass( 9) = 59;      %XIAP bound to the p2-fragment
mass(10) = 89;      %XIAP bound to the p2-fragment and active Caspase 3
mass(11) = 28;      %XIAP fragment of domains BIR1 and BIR2
mass(12) = 29;      %XIAP fragment of domains BIR3 and RING
mass(13) = 58;      %BIR12 bound to Caspase 3
mass(14) = 729;     %BIR3R bound to Caspase 9 (p35/p12) bound to the apoptosome
mass(15) = 31;      %BIR3R bound to the p2-fragment
mass(16) = 27;      %free SMAC
mass(17) = 111;     %XIAP bound to 2SMAC
mass(18) = 86;      %XIAP bound to the p2-fragment and SMAC
mass(19) = 55;      %BIR12 bound to 2SMAC
mass(20) = 56;      %BIR3R bound to SMAC
mass(21) = 54;      %FRET substrate

%
pagfpmass       = 27;                       %    Reference diffusion coefficient of PAGFP in uM**2 * min**(-1)
k_space         = t12propagation/xmax;      %    propagation constant of signal: exp(-kspace*(x-x0))
ix0             = 0;                        %    spatial starting point of the signal

% if idiff ==1
%    'Apoptosome dependent items blocked'
% else
%    'Stokes-Einstein movement of all entities'
% end

for i = 1:npartners-2
    diff(i) = pagfpd*(pagfpmass/mass(i))^(1/3); % calculate diffusion rate from reference

    if idiff ==1 && mass(i)>500
        diff(i) =0;
    end
end
%
%       Input function maintain spatial gradient:%
%       The input signal is  trigger information
%
diff(21) = 0;
% (substrate is fixed)
diff(22) = 0;
% (spatial gradient in input function 1)
diff(23) = 0;
% (spatial gradient in input function 2)
%
kp(3:6)      = kp(3:6)*x_enzyme;
kp(11:20)    = kp(11:20)*x_enzyme;
km(1:2)      = kp(1:2)*x_enzyme;
kp(7)        = kp(7)*x_enzyme;
kp(10)       = kp(10)*x_enzyme;
kp(21)       = kp(21)*x_enzyme;
kp(23)       = kp(23)*x_enzyme;
kp(26:34)    = kp(26:34)*x_enzyme;

km(7)        = km(7)*x_enzyme;
km(10)       = km(10)*x_enzyme;
km(21)       = km(21)*x_enzyme;
km(23)       = km(23)*x_enzyme;
km(26:34)    = km(26:34)*x_enzyme;


%%%%%%%%%%%%%%%%%%%%%%%%%%%%%%%%%%%%%%%%%%%%%%%%%%%%%%%%%%%%%%%%%%%%%%%%%%%%%%%%%%%%%%%
% Normation values
%%%%%%%%%%%%%%%%%%%%%%%%%%%%%%%%%%%%%%%%%%%%%%%%%%%%%%%%%%%%%%%%%%%%%%%%%%%%%%%%%%%%%%%%

xnorm       = ones(23,1);
xnorm(1)    = PCAS3;
xnorm(2)    = a0;
xnorm(3)    = PCAS3;
xnorm(4)    = a0;
xnorm(5:15) = XIAP;
xnorm(16)   = smac0;
xnorm(17)   = smac0;
xnorm(18:20)= smac0;
xnorm(21)   = 1;
xnorm(22)   = a0;
xnorm(23)   = smac0;

%%%%%%%%%%%%%%%%%%%%%%%%%%%%%%%%%%%%%%%%%%%%%%%%%%%%%%%%%%%%%%%%%%%%%%%%%%%%%%%%%%%%%%%
% S- Matrix (for definition and purpose, see also Rehm 2006)
%
% The S-Matrix translates reaction stoichiometry into a Matrix formalism
% e.g. reaction 4:  C9a	 +	C3a    -->     C9H	+	C3a leads to
%
%                   S(2,4)      =   -1 (C9a is lost)
%                   S(3,4)      =   0  (C3a remains unchanged)
%                   S(4,4)      =   1  (C9a is produced)
%                   S(other,4)  =   0  (not used)
%
%
% Syntax: S(reaction partner, reaction)
%
%%%%%%%%%%%%%%%%%%%%%%%%%%%%%%%%%%%%%%%%%%%%%%%%%%%%%%%%%%%%%%%%%%%%%%%%%%%%%%%%%%%%%%%%

S               = zeros(npartners,nreactions);      %   S- matrix
vel             = zeros(nreactions*6,1);            %   velocity pointer, see below

% Production for C3

S(1,1)  =    1;
S(1,3)  =   -1;
S(1,5)  =   -1;
S(1,6)  =   -1;

% Production for C9

S(2,4)  =   -1;
S(2,21) =   -1;
S(2,22) =   -1;
S(2,23) =   -1;
S(2,27) =    1;
S(2,29) =    1;
S(2,33) =    1;
S(2,36) =   -1;

% Production for C9*

S(3,3)  =    1;
S(3,5)  =    1;
S(3,6)  =    1;
S(3,7)  =   -1;
S(3,8)  =   -1;
S(3,9)  =   -1;
S(3,10) =   -1;
S(3,28) =    1;
S(3,29) =    1;
S(3,32) =    1;
S(3,37) =   -1;

% Production for C9**

S(4,4)  =    1;
S(4,18) =    1;
S(4,19) =    1;
S(4,20) =    1;
S(4,35) =   -1;

% Production for XIAP

S(5,2)  =   1; % XIAP turnover rate
S(5,7)  =   -1;
S(5,11) =   -1;
S(5,21) =   -1;
S(5,25) =   1;
S(5,26) =   -1;

% Production for [XIAP~C3*]

S(6,7)      =1;
S(6,13)     =-1;
S(6,22)     =-1;
S(6,28)     =-1;
S(6,38)     =-1;

% Production for [XIAP~C9*]

S(7,8)      =-1;
S(7,12)     =-1;
S(7,19)     =-1;
S(7,21)     =1;
S(7,27)     =-1;
S(7,40)     =-1;

% Production for [XIAP~C9*~C3*]

S(8,8)      =1;
S(8,16)     =-1;
S(8,18)     =-1;
S(8,22)     =1;
S(8,29)     =-1;
S(8,39)     =-1;

% Production for [XIAP~{cl-C9*}]

S(9,9)      =-1;
S(9,14)     =-1;
S(9,19)     =1;
S(9,25)     =-1;
S(9,34)     =-1;
S(9,41)     =-1;

% Production for [[XIAP~{cl-C9*}]~C3*]

S(10,9)     =1;
S(10,15)    =-1;
S(10,18)    =1;
S(10,42)    =-1;

% Production for BIR12

S(11,10)    =-1;
S(11,11)    =1;
S(11,12)    =1;
S(11,14)    =1;
S(11,30)    =-1;
S(11,45)    =-1;

% Production for BIR3R

S(12,11)    =1;
S(12,13)    =1;
S(12,23)    =-1;
S(12,24)    =1;
S(12,31)    =-1;
S(12,46)    =-1;

% Production for [BIR12~C3*]

S(13,10)    =1;
S(13,13)    =1;
S(13,15)    =1;
S(13,16)    =1;
S(13,32)    =-1;
S(13,49)    =-1;

% Production for [BIR3R~C9*]

S(14,12)    =1;
S(14,16)    =1;
S(14,20)    =-1;
S(14,23)    =1;
S(14,33)    =-1;
S(14,50)    =-1;

% Production for [BIR3R~{cl-C9*}]

S(15,14)    =1;
S(15,15)    =1;
S(15,20)    =1;
S(15,24)    =-1;
S(15,51)    =-1;

% Production for SMAC

S(16,26)    =-2;
S(16,27)    =-2;
S(16,28)    =-2;
S(16,29)    =-2;
S(16,30)    =-1;
S(16,31)    =-1;
S(16,32)    =-1;
S(16,33)    =-1;
S(16,34)    =-2;
S(16,52)    =-1;

% Production for [XIAP~2SMAC]

S(17,17)    =-1;
S(17,26)    =1;
S(17,27)    =1;
S(17,28)    =1;
S(17,29)    =1;
S(17,44)    =-1;

% Production for [[XIAP~{cl-C9*}]~  SMAC]

S(18,34)    =1;
S(18,43)    =-1;

% Production for [BIR12~SMAC]

S(19,17)    =1;
S(19,30)    =1;
S(19,32)    =1;
S(19,47)    =-1;

% Production for [BIR3R~SMAC]

S(20,17)    =1;
S(20,31)    =1;
S(20,33)    =1;
S(20,48)    =-1;

% Production for Substrate

S(21,53)    = -1;

%%%%%%%%%%%%%%%%%%%%%%%%%%%%%%%%%%%%%%%%%%%%%%%%%%%%%%%%%%%%%%%%%%%%%%%%%%%%%%%%%%%%%%%
% Dynamic Input functions
%%%%%%%%%%%%%%%%%%%%%%%%%%%%%%%%%%%%%%%%%%%%%%%%%%%%%%%%%%%%%%%%%%%%%%%%%%%%%%%%%%%%%%%%

S(2,54)     =  1;
S(22,54)    = -1;
S(16,55)    =  1;
S(23,55)    = -1;
%
%%%%%%%%%%%%%%%%%%%%%%%%%%%%%%%%%%%%%%%%%%%%%%%%%%%%%%%%%%%%%%%%%%%%%%%%%%%%%%%%%%%%%%%%


%%%%%%%%%%%%%%%%%%%%%%%%%%%%%%%%%%%%%%%%%%%%%%%%%%%%%%%%%%%%%%%%%%%%%%%%%%%%%%%%%%%%%%%
%
% The field "vel" is used for calculation of the velocity vector
%
% Indirect addressing is used to calculate each velocity,
% e.g. 2 1 24 24 24 24 in the second row means
% v2 = k2p*c2*c1*c24 - k2m*c2*c1*c24
% with c24 = 1 and k2m = 0 this leads to v2 = k2p*c2*c1.
% (c1 ..cn are defined in the convention section)
%
% General:  each row denotes one reaction
%           first 3 elements of each row for forward
%           last 3 for backward reaction
%           forward and backward reaction are multiplied by the respective
%           kinetic constant
%           first two reactions are structurally different.
%
% Further examples:
%
% e.g. : v1 = kp(1)*1 * 1 * 1 - km(1)*c1*1*1*1 (c1= C3, see above)
% e.g. : v2 = kp(2) - km(2)*c5 (C5= XIAP, see above)
% e.g. : v3 = kp(3)*c2*c1- 0*1*1*1 (= kp(3) * C3 * C9*)
%
%%%%%%%%%%%%%%%%%%%%%%%%%%%%%%%%%%%%%%%%%%%%%%%%%%%%%%%%%%%%%%%%%%%%%%%%%%%%%%%%%%%%%%%%


vel = [24 24 24 1 24 24 ... % turnover -> C3_
    24 24 24 5 24 24  ...   % turnover -> XIAP
    2  1 24 24 24 24  ...   % C9a	+	C3_                     -->     C9a	+	C3a
    2  3 24 24 24 24  ...   % C9a	+	C3a                     -->     C9H	+	C3a
    4  1 24 24 24 24 ...    % C9H	+	C3_                     -->     C9H	+	C3a
    1  3 24 24 24 24  ...   % C3_	+	C3a                     -->     C3a	+	C3a
    3  5 24  6 24 24 ...    % C3a	+	XIAP_                   < = >	[XIAP~C3a]
    3  7 24  8 24 24 ...    % C3a	+	[XIAP~C9a]              < = >	[XIAP~C9a~C3a]
    3  9 24 10 24 24 ...    % C3a	+	[XIAP~{cl-C9a}]         < = >	[[XIAP~{cl-C9a}]~C3a]
    3 11 24 13 24 24 ...    % C3a	+	BIR12_                  < = >	[BIR12~C3a]
    3  5 24 24 24 24 ...    % C3a	+	XIAP_                   -->     BIR12_	+	BIR3R_	+	C3a
    3  7 24 24 24 24 ...    % C3a	+	[XIAP~C9a]              -->     BIR12_	+	[BIR3R~C9a]	+	C3a
    3  6 24 24 24 24 ...    % C3a	+	[XIAP~C3a]              -->     C3a     +	[BIR12~C3a]	+	BIR3R_
    3  9 24 24 24 24 ...    % C3a	+	[XIAP~{cl-C9a}]         -->     C3a     +	BIR12_	+	[BIR3R~{cl-C9a}]
    3 10 24 24 24 24  ...   % C3a	+	 [[XIAP~{cl-C9a}]~C3a]	-->     C3a     +	[BIR12~C3a]	+	[BIR3R~{cl-C9a}]
    3  8 24 24 24 24 ...    % C3a	+	[XIAP~C9a~C3a]			-->     C3a     +	[BIR12~C3a]	+	[BIR3R~C9a]
    3 17 24 24 24 24 ...    % C3a	+	[XIAP~2SMAC]			-->     C3a     +	[BIR12~SMAC]	+	[BIR3R~SMAC]
    3  8 24 24 24 24 ...    % C3a	+	[XIAP~C9a~C3a]			-->     C3a     +	C9H	+	 [[XIAP~{cl-C9a}]~C3a]
    3  7 24 24 24 24 ...    % C3a	+	[XIAP~C9a]              -->     C3a     +	C9H	+	[XIAP~{cl-C9a}]
    3 14 24 24 24 24  ...   % C3a	+	[BIR3R~C9a]             -->     C3a     +	[BIR3R~{cl-C9a}]	+	C9H
    2  5 24  7 24 24 ...    % C9a	+	XIAP_                   < = >	[XIAP~C9a]
    2  6 24  8 24 24 ...    % C9a	+	[XIAP~C3a]              < = >	[XIAP~C9a~C3a]
    2 12 24 14 24 24 ...    % C9a	+	BIR3R_                  < = >	[BIR3R~C9a]
    15 24 24 24 24 24  ...  % [BIR3R~{cl-C9a}]					-->     BIR3R_
    9 24 24 24 24 24 ...    % [XIAP~{cl-C9a}]					-->     XIAP_
    5 16 16 17 24 24 ...    % XIAP_	+	SMAC_	+	SMAC_       < = >	[XIAP~2SMAC]
    7 16 16 17  2 24 ...    % [XIAP~C9a]	+	SMAC_	+ SMAC_	< = >	[XIAP~2SMAC]	+	C9a
    6 16 16 17  3 24  ...   % [XIAP~C3a]	+	SMAC_	+ SMAC_	< = >	[XIAP~2SMAC]	+	C3a
    8 16 16 17  3  2  ...   % [XIAP~C9a~C3a]+	SMAC_+	SMAC_	< = >	[XIAP~2SMAC]	+	C3a	+	C9H
    11 16 24 19 24 24  ...  % BIR12_	+	SMAC_    			< = >	[BIR12~SMAC]
    12 16 24 20 24 24 ...   % BIR3R_	+	SMAC_			    < = >	[BIR3R~SMAC]
    13 16 24 19  3 24 ...   % [BIR12~C3a]	+	SMAC_			< = >	[BIR12~SMAC]	+	C3a
    14 16 24 20  2 24 ...   % [BIR3R~C9a]	+	SMAC_			< = >	[BIR3R~SMAC]	+	C9a
    9 16 24 18 24 24 ...    % [XIAP~{cl-C9a}]	+	SMAC_+SMAC_	< = >	[XIAP~{cl-C9a}]~  SMAC]
    4 24 24 24 24 24 ...    % C9H                               -->
    2 24 24 24 24 24 ...    % C9a                               -->
    3 24 24 24 24 24 ...    % C3a                               -->
    6 24 24 24 24 24  ...   % [XIAP~C3a]                        -->
    8 24 24 24 24 24  ...   % [XIAP~C9a~C3a]                    -->
    7 24 24 24 24 24  ...   % [XIAP~C9a]                        -->
    9 24 24 24 24 24 ...    % [XIAP~{cl-C9a}]                   -->
    10 24 24 24 24 24 ...   % [[XIAP~{cl-C9a}]~C3a]             -->
    18 24 24 24 24 24  ...  % [XIAP~{cl-C9a}]~  SMAC]           -->
    17 24 24 24 24 24  ...  % [XIAP~2SMAC]                      -->
    11 24 24 24 24 24 ...   % BIR12_                            -->
    12 24 24 24 24 24  ...  % BIR3R_                            -->
    19 24 24 24 24 24 ...   % [BIR12~SMAC]                      -->
    20 24 24 24 24 24 ...   % [BIR3R~SMAC]                      -->
    13 24 24 24 24 24  ...  % [BIR12~C3a]                       -->
    14 24 24 24 24 24 ...   % [BIR3R~C9a]                       -->
    15 24 24 24 24 24 ...   % [BIR3R~{cl-C9a}]                  -->
    16 24 24 24 24 24 ...   % SMAC_                             -->
    21 03 24 24 24 24 ...    % Substrate  (by C3)               -->
    22 24 24 24 24 24 ...   %APAF/C9 + no restricting           -->		Apoptosome (leads to exponential saturation)
    23 24 24 24 24 24 ];    %SMACmito                           -->		SMAC   (leads to exponential saturation)

%%%%%%%%%%%%%%%%%%%%%%%%%%%%%%%%%%%%%%%%%%%%%%%%%%%%%%%%%%%%%%%%%%%%%%%%%%%%%%%%%%%%%%%
% Reference vector for Jacobi Matrix jac = jac (74*6, 26)

% pairs of 6 - 3 for forward, 3 for backward, 27 = dummy1(=1),
% 25 =dummy2(=0), 26 = dummy3 (=2), 27 = dummy4 (=3)
%
%%%%%%%%%%%%%%%%%%%%%%%%%%%%%%%%%%%%%%%%%%%%%%%%%%%%%%%%%%%%%%%%%%%%%%%%%%%%%%%%%%%%%%%%

kp( 1)      = PCAS3*km(1); %kp(1)/km(1) = Caspase 3 concentration
kp( 2)      = XIAP*km(2);  %kp(2)/km(2) = XIAP concentration

xo          = zeros(23,1);
xo (1)      = PCAS3;                % Procaspase 3
xo (3)      = 0.;
xo (5)      = XIAP;                 % XIAP
xo (21)     = 1;                    % Substrate (dynamic calculation)
xo (22)     = a0;                   % inactive APAF1 -> gets converted to active APAF1
xo (23)     = smac0;                % mitochondrial SMAC -> cytosolic SMAC
%
%
switch IMODE
    case {0,1,2,4}
    %
    %
    % Calculations with single diffusion coefficient
    %
    %
    %%%%%%%%%%%%%%%%%%%%%%%%%%%%%%%%%%%%%%%%%%%%%%%%%%%%%%%%%%%%%%%%%%%%%%%%%%%%%%%%%%%%%%%
    % Call of Runge-Kutta Iteration (begin)
    %%%%%%%%%%%%%%%%%%%%%%%%%%%%%%%%%%%%%%%%%%%%%%%%%%%%%%%%%%%%%%%%%%%%%%%%%%%%%%%%%%%%%%%
    %
    Conc       = pdepe(0, @pde2, @init2, @bc2, xlist, tlist);
    %
    %%%%%%%%%%%%%%%%%%%%%%%%%%%%%%%%%%%%%%%%%%%%%%%%%%%%%%%%%%%%%%%%%%%%%%%%%%%%%%%%%%%%%%%
    %
    [j,isize]   = size(tlist);
    [j,jsize]   = size(xlist);

    C2 = zeros (isize,23);

    for i = 1:npartners
        for j = 1:jsize
            C2(:,j,i) =  Conc(:,j,i) / xnorm(i); % Normalize constants
        end
    end

    %%%%%%%%%%%%%%%%%%%%%%%%%%%%%%%%%%%%%%%%%%%%%%%%%%%%%%%%%%%%%%%%%%%%%%%%%%%%%%%%%%%%%%%
    % Output of Results into figures
    %%%%%%%%%%%%%%%%%%%%%%%%%%%%%%%%%%%%%%%%%%%%%%%%%%%%%%%%%%%%%%%%%%%%%%%%%%%%%%%%%%%%%%%%

    for i = 1:jsize
        Sub(:,i)             = C2(:,i,21);
        Casp3(:,i)           = C2(:,i,3);
        Apop(:,i)            = 1 - C2(:,i,22);
        Smac(:,i)            = 1 - C2(:,i,23);
    end
    %
    % cleavage profiles at begin and end
    %
    Sub_begin               = Sub(:,1);
    Sub_end                 = Sub(:,jsize);
    Apop_begin              = Apop(:,1);
    Apop_end                = Apop(:,jsize);
    Smac_begin              = Smac(:,1);
    Smac_end                = Smac(:,jsize);

    if IMODE ==0
    %%%%%%%%%%%%%%%  Figure 2 a (IMODES = 0) %%%%%%%%%%
    figure
    plot(tlist,100*Apop_begin, tlist,100*Apop_end)
    hold on
    set(gca,'FontSize',8)
    set(gca,'FontName','Arial')
    axis([0,tmax,0,100])
    set (gca, 'LineWidth',2)
    xlabel('Time after model initiation [min]')
    ylabel('Input of cyt-c induced apoptosome formation [%]')
    set (gca, 'LineWidth',2)
    legend('near end','far end')

    %%%%%%%%%%%%%%%  Figure 2 b (IMODES = 0) %%%%%%%%%%
    figure
    plot(tlist,100*Smac_begin, tlist,100*Smac_end)
    hold on
    set(gca,'FontSize',8)
    set(gca,'FontName','Arial')
    axis([0,tmax,0,100])
    set (gca, 'LineWidth',2)
    xlabel('Time after model initiation [min]')
    ylabel('SMAC input [%]')
    set (gca, 'LineWidth',2)
    legend('near end','far end')
    end

    if IMODE == 0 || IMODE == 1
    %%%%%%%%%%%%%%%  Figure 2 c (IMODES 0 and 1) %%%%%%%%%%
    figure
    mesh(xlist, tlist,100*Apop(:,:), 'LineStyle','none', 'Marker','.', 'MarkerSize', 6)
    hold on
    set(gca,'FontSize',12)
    set(gca,'FontName','Arial')
    axis([0,max(xlist),0,max(tlist),0,100])
    xlabel('Distance from point of onset [um]')
    ylabel('Time after model initiation [min]')
    zlabel('Apoptosome formation [%]')
    set (gca, 'LineWidth',2)
    set(gca,'XTick',0:5:max(xlist))
    set(gca,'YTick',0:5:max(tlist))
    set(gca,'ZTick',0:20:100)
    view (0,90)
    %
    %
    %%%%%%%%%%%%%%%  Figure 2 d (IMODES 0 and 1) %%%%%%%%%%
    figure
    mesh(xlist, tlist,100*Smac(:,:), 'LineStyle','none', 'Marker','.', 'MarkerSize', 6)
    hold on
    set(gca,'FontSize',12)
    set(gca,'FontName','Arial')
    axis([0,max(xlist),0,max(tlist),0,100])
    xlabel('Distance from point of onset [um]')
    ylabel('Time after model initiation [min]')
    zlabel('Local Smac influx [%]')
    set(gca, 'LineWidth',2)
    set(gca,'XTick',0:50:max(xlist))
    set(gca,'YTick',0:10:max(tlist))
    set(gca,'ZTick',0:20:100)
    view (0,90)
   %
    %%%%%%%%%  Figure 3 c,d (c:IMODE=1, d:IMODES = 0)   %%%%%%%%%%%%%%
    figure
    mesh(xlist, tlist,100*(1-Sub(:,:)),'LineStyle','none', 'Marker','.', 'MarkerSize', 6)
    hold on
    set(gca,'FontSize',12)
    set(gca,'FontName','Arial')
    axis([0,max(xlist),0,max(tlist),0,100])
    xlabel('Distance from point of onset [um]')
    ylabel('Time after model initiation [min]')
    zlabel('Substrate Cleavage [%]')
    set (gca, 'LineWidth',2)
    set(gca,'XTick',0:5:max(xlist))
    set(gca,'YTick',0:5:max(tlist))
    set(gca,'ZTick',0:20:100)
    view (0,90)

    %%%%%%%%%  Figure 3 c,d (c:IMODE=1, d:IMODES = 0)   %%%%%%%%%%%%%%
    figure
    mesh(xlist, tlist,100*Casp3(:,:), 'LineStyle','none', 'Marker','.', 'MarkerSize', 6)
    hold on
    set(gca,'FontSize',12)
    set(gca,'FontName','Arial')
    axis([0,max(xlist),0,max(tlist),0,100])
    xlabel('Distance from point of onset [um]')
    ylabel('Time after model initiation [min]')
    zlabel('free active Caspase [%]')
    set(gca, 'LineWidth',2)
    set(gca,'XTick',0:5:max(xlist))
    set(gca,'YTick',0:5:max(tlist))
    set(gca,'ZTick',0:20:100)
    view (0,90)
    end

    if IMODE ==2
    %%%  Figure 6 c,d,e,f (IMODE=2, downfact = 10,100,1000,10000) %%%%
    figure
    plot(tlist,100*Sub_begin, tlist,100*Sub_end)   % Fig 3a, Caspases and input functions
    hold on
    set(gca,'FontSize',8)
    set(gca,'FontName','Arial')
    axis([0,max(tlist),0,100])
    set (gca, 'LineWidth',2)
    xlabel('Time after model initiation [min]')
    ylabel('Substrate cleavage [%]')
    set (gca, 'LineWidth',2)
    legend('near end','far end')
    end


    [j,isize]   = size(tlist);

    for j = 1:isize
        if 1-Sub_begin(j)>=stest1
            t00 = tlist(j);
            break
        end
    end

    for j = 1:isize
        if 1-Sub_end(j)>=stest1
            t01 = tlist(j);
            break
        end
    end
%
    for j = 1:isize
        if 1-Sub_begin(j)>=stest2
            t10 = tlist(j);
            break
        end
    end

    for j = 1:isize
        if 1-Sub_end(j)>=stest2
            t11 = tlist(j);
            break
        end
    end
    it1 = t01 - t00;
    it2 = t11 - t10;
    %
    %
case {3}
    %
    %
    % Regulation studies of diffusion coefficient
    %
    %
    itest       = 100;
    jcheck      = 99;
    check       = zeros(1,jcheck);
    xin         = zeros(itest,1);
    xmax1       = 1;
    xmin1       = 1./10000;
    lxmin1      = log10(xmin1);
    lxmax1      = log10(xmax1);

    [j,isize]   = size(tlist);
    submem0 = zeros(isize, itest);
    submem1 = zeros(isize, itest);


    for it = 1:itest+1
        %
        it  % Print out iteration step
        %
        % get logarithmic mesh points
        %
        xi          = lxmin1 +  (lxmax1 -lxmin1)/itest *(it-1);
        xin(it)     = 10^xi;
        xmem        = xin(it);
        xmem
        pagfpd      = 24*60*xin(it);            % Modulated diffusion coefficients
        pagfpmass   = 27;                       % Reference diffusion coefficients of PAGFP in uM**2 * min**(-1)
        k_space     = t12propagation/xmax;      % propagation constant of signal: exp(-kspace*(x-x0))


        for i = 1:npartners-2
            diff(i) = pagfpd*(pagfpmass/mass(i))^(1/3); % calculate diffusion rate from reference
        end
        diff(21) = 0;
        diff(22) = 0;
        diff(23) = 0;
        %
        [j,jsize]   = size(xlist);
        %
        Conc        = pdepe(0, @pde2, @init2, @bc2, xlist, tlist);
        Subx0       = 1-Conc(:,1,21);       % Substrate cleavage kinetics at x =  0
        Subxm       = 1-Conc(:,jsize,21);   % Substrate cleavage kinetics at x = 30

        Submem0(:,it) = Subx0;
        Submem1(:,it) = Subxm;

        %
        % check time for 1,2,3,4, ...  substrate cleavage at x =  0
        %
        for i = 1:jcheck
            check(i)  = i/100;
        end
        %
        ix0         = ones(jcheck,1);
        ixm         = ones(jcheck,1);
        j           = 1;
        i           = 1;
        %
        % check time for 1,2,3,4, ...  substrate cleavage at x =  30
        %
        while (j<jcheck+1)
            i = i +1;
            if Subx0(i)>check(j)
                ix0(j) = i;
                j = j+1;
            end
        end
        %
        j = 1;
        i = 1;
        while (j<jcheck+1)
            i = i +1;
            if Subxm(i)>check(j)
                ixm(j) = i;
                j = j+1;
            end
        end
        %
        % and get the difference
        %
        for j = 1: jcheck
            delaysub(it,j) = tlist(ixm(j)) - tlist(ix0(j));
        end
    end
%
    save('test', 'check', 'xin', 'delaysub', 'xin')
%
%   The following routine is to provide a colormap for a set of x,y-graphs
%
iin     = jcheck-1;         % 99 output lines
iref    = 64;              % 64 colorbar maps
%
npoints = 5;
cindex  = zeros(5,4);
%
%   Colourmap RGB values taken from MATLAB
%

cindex(1,:) = [ 1 0     0 255];     % Index array: Index, R G B
cindex(2,:) = [24 0   255 255];     % for 5 colourmap marker
cindex(3,:) = [40 255 255   0];     % Therefore, we have five intervals
cindex(4,:) = [56 255   0   0];     % which we have to interpolate
cindex(5,:) = [64 128   0   0];

ipoints     = 1;
iold        = 0;

figure
for i = 1:iin

    inew = cindex(ipoints+1,1)*iin/iref;    % right side marker point
    if i>inew;                              % Is current index already in the next interval
        iold    =   i-1;
        ipoints =   ipoints +1;
        inew    =   cindex(ipoints+1,1)*iin/iref;
    end
if ipoints == npoints
    break
end
    distance    = (i-iold)/(inew-iold);
    col(1)      = cindex(ipoints,2)+ (cindex(ipoints+1,2) -cindex(ipoints,2))*distance;
    col(2)      = cindex(ipoints,3)+ (cindex(ipoints+1,3) -cindex(ipoints,3))*distance;
    col(3)      = cindex(ipoints,4)+ (cindex(ipoints+1,4) -cindex(ipoints,4))*distance;
    plot(xin(:),delaysub(:,i),'Color',col/255, 'LineWidth',2)
    hold on
end
    %
    % Fig 6b output (IMODE =3)
    %
    set(gca,'FontSize',12)
    set(gca,'FontName','Arial')
    set(gca,'XScale','log')
    axis([0.0001,1,0,6])
    xlabel('Rel. change of parameter')
    ylabel('End to end difference to 1, 50 or 80 % substrate cleavage [min]')
    legend( '100% substrate cleavage: red, 0 %substrate cleavage: blue' )

end

end


function [c_t, f, res]  = pde2(x,t,c,dcdx)
global km kp S vel sbreakdown diff nreactions
global k_space
%
%
[j,isize]   = size(dcdx);
c_t         = ones(j,1);
%
% Flux term (f)
for i= 1:j
    f(i,1) = diff(i)*dcdx(i);
end
% Diffusion equation in slab geometry initial conditions (zero on boundary)
%
% Solving:
%   c u_t = f_x(u,u_x) + s(u,u_x)
%
v=zeros(nreactions,1);
c(24) = 1; %Dummy1
% Indirect adressing vector dc/dt = K+ *conc1*conc2*conc3 - K- *conc4*conc5*conc6
if c(21) < sbreakdown       % after this amount of substrate left,
    % no more protein production and no enforced degradation
    kp(1:2) = 0;            % Breakdown of Protein production
    km(1:2) = 0;            % Breakdown of Protein production
    kp(38:39)   = 0.0058;   % No enforced degradation
    kp(40)      = 0.0058;
    kp(43:44)   = 0.0058;
    kp(46)      = 0.0058;
    kp(48)      = 0.0058;
    kp(50:51)   = 0.0058;
    kp(55:56)   = 0.0058;
end
%
% Here, we create an delay in the input function to mimic the spatial
% propagation of the initiator signal. The delay is modelled by the logical
% expression (t >= k_space*x). This means that the input at the spatial point x
% is started at the time point t = k_space*x
%
% Note: an exponential saturation function (Apoptosome formation, Smac
% release) can be mimicked by a first order chemical equation. The
% pseudo-equations are: inactive APAF-1 -> active apoptosome spoke (with
% Casp-9) and Smac(mito) -> Smac(cyto).
%
for i = 1:nreactions
    v(i) = kp(i)*c(vel(6*i-5))*c(vel(6*i-4))*c(vel(6*i-3)) - ....
        km(i)*c(vel(6*i-2))*c(vel(6*i-1))*c(vel(6*i-0));

    % Indirect addressing vector dc/dt = K+ *conc1*conc2*conc3 - K- *conc4*conc5*conc6

    if i == 54
        v(i) = v(i)*(t >= k_space*x);   % delay: t_delay(x) = k_space*x = t12propagation*x/xmax;
    end
    if  i == 55
        v(i) = v(i)*(t >= k_space*x);
    end
end
%
% next iteration step
%
res = S*v;
%
end
%

%%%%%%%%%%%%%%%%%%%%%%%%%%%%%%%%%%%%%%%%%%%%%%%%%%%%%%%%%%%%%%%%%%%%%%%%%%%%%%%%%%%%%%%
% Input functions
%%%%%%%%%%%%%%%%%%%%%%%%%%%%%%%%%%%%%%%%%%%%%%%%%%%%%%%%%%%%%%%%%%%%%%%%%%%%%%%%%%%%%%%%

function [pl, ql, pr, qr] = bc2(xl, ul, xr, ur, t);
pl = zeros(size(ul));
ql = ones(size(ul));
pr = zeros(size(ur));
qr = ones(size(ur));

% PDE definition for diffusion-reaction equation in slab geometry
%
% Boundary conditions of Neumann type: vanishing derivative at slab ends
%
% Solving:
%   c u_t = f_x(u,u_x) + s(u,u_x)
end

function val = init2(x)
global xo
val = xo;
% Initial condition with homogenous distribution of proteins
end
```

Published with MATLAB® 7.5
