# Supplementary material for: Diffusion is capable of translating anisotropic apoptosis initiation into a homogeneous execution of cell death
Source: BMC Syst Biol. 2010 Feb 4;4:9. doi: 10.1186/1752-0509-4-9 (PMC2831829; doi:10.1186/1752-0509-4-9)
Supplement: Additional file 6 — Photobleaching control measurement for rapid FRET sampling. Fluorescence signal intensities in CFP, FRET, and YFP channels were measured in unstimulated cells at rapid sampling conditions. This control excludes that photodamage by the acquisition process influenced the experimental measurements of effector caspase activation. [file 1752-0509-4-9-S6.PDF]

Additional File 6

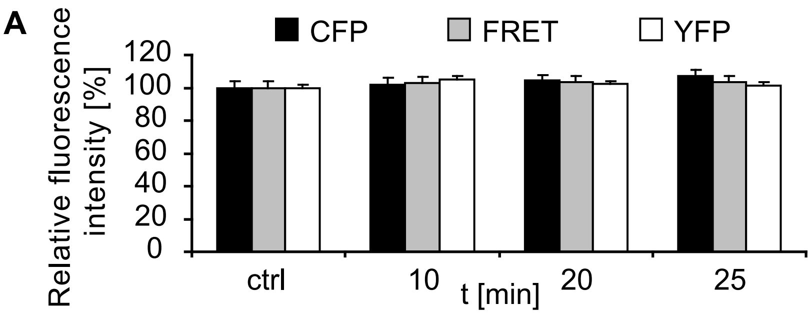

Figure Legend Additional File 6: Fast sampling during DEVDase FRET measurements does not result in photobleaching.

Cellular fluorescence in the CFP, FRET and YFP channels during the first minute of fast sampling was scaled to 100 % and compared to the average cellular fluorescence during minutes 10, 20, and 25. Data show means + s.d. from n = 8 untreated DEVDase FRET probe expressing HeLa cells which were confocally scanned at intervals of 4 sec.
